# Functional Genetic Elements for Controlling Gene Expression in Cupriavidus necator H16

**DOI:** 10.1128/AEM.00878-18

**Published:** 2018-09-17

**Authors:** Swathi Alagesan, Erik K. R. Hanko, Naglis Malys, Muhammad Ehsaan, Klaus Winzer, Nigel P. Minton

**Affiliations:** a BBSRC/EPSRC Synthetic Biology Research Centre, School of Life Sciences, Centre for Biomolecular Sciences, The University of Nottingham, Nottingham, United Kingdom; University of Buenos Aires

**Keywords:** promoter, ribosome binding site, functional genetic element, gene expression, isoprene, Cupriavidus necator H16

## Abstract

A robust and predictable control of gene expression plays an important role in synthetic biology and biotechnology applications. Development and quantitative evaluation of functional genetic elements, such as constitutive and inducible promoters as well as ribosome binding sites (RBSs), are required. In this study, we designed, built, and tested promoters and RBSs for controlling gene expression in the model lithoautotroph Cupriavidus necator H16. A series of variable-strength, insulated, constitutive promoters exhibiting predictable activity within a >700-fold dynamic range was compared to the native P_*phaC*_, with the majority of promoters displaying up to a 9-fold higher activity. Positively (AraC/P_*araBAD*_-l-arabinose and RhaRS/P_*rhaBAD*_-l-rhamnose) and negatively (AcuR/P_*acuRI*_-acrylate and CymR/P_*cmt*_-cumate) regulated inducible systems were evaluated. By supplying different concentrations of inducers, a >1,000-fold range of gene expression levels was achieved. Application of inducible systems for controlling expression of the isoprene synthase gene *ispS* led to isoprene yields that exhibited a significant correlation to the reporter protein synthesis levels. The impact of designed RBSs and other genetic elements, such as mRNA stem-loop structure and A/U-rich sequence, on gene expression was also evaluated. A second-order polynomial relationship was observed between the RBS activities and isoprene yields. This report presents quantitative data on regulatory genetic elements and expands the genetic toolbox of C. necator.

**IMPORTANCE** This report provides tools for robust and predictable control of gene expression in the model lithoautotroph C. necator H16. To address a current need, we designed, built, and tested promoters and RBSs for controlling gene expression in C. necator H16. To answer a question on how existing and newly developed inducible systems compare, two positively (AraC/P_*araBAD*_-l-arabinose and RhaRS/P_*rhaBAD*_-l-rhamnose) and two negatively (AcuR/P_*acuRI*_-acrylate and CymR/P_*cmt*_-cumate) regulated inducible systems were quantitatively evaluated and their induction kinetics analyzed. To establish if gene expression can be further improved, the effect of genetic elements, such as mRNA stem-loop structure and A/U-rich sequence, on gene expression was evaluated. Using isoprene production as an example, the study investigated if and to what extent chemical compound yield correlates to the level of gene expression of product-synthesizing enzyme.

## Abstract (precis)

This report provides tools for robust and predictable control of gene expression in the model lithoautotroph C. necator H16. To address a current need, we designed, built, and tested promoters and RBSs for controlling gene expression in C. necator H16. To answer a question on how existing and newly developed inducible systems compare, two positively (AraC/P_*araBAD*_-l-arabinose and RhaRS/P_*rhaBAD*_-l-rhamnose) and two negatively (AcuR/P_*acuRI*_-acrylate and CymR/P_*cmt*_-cumate) regulated inducible systems were quantitatively evaluated and their induction kinetics analyzed. To establish if gene expression can be further improved, the effect of genetic elements, such as mRNA stem-loop structure and A/U-rich sequence, on gene expression was evaluated. Using isoprene production as an example, the study investigated if and to what extent chemical compound yield correlates to the level of gene expression of product-synthesizing enzyme.

## INTRODUCTION

Cupriavidus necator H16 is a Gram-negative betaproteobacterium, formerly known as Ralstonia eutropha, Alcaligenes eutrophus, Wautersia eutropha, and Hydrogenomonas eutropha. It has been extensively studied for its capacity to store large amounts of organic carbon in the form of poly-3-hydroxybutyrate (PHB) that can be utilized as a source of bioplastics (1, 2). Importantly, this chemolithoautotrophic bacterium possesses the ability to use both organic compounds and molecular hydrogen (H_2_) as sources of energy, utilizing them to power metabolic processes and fix carbon dioxide (CO_2_) (3). In the absence of oxygen (O_2_), C. necator H16 can switch to anaerobic respiration-denitrification, exploiting alternative electron acceptors such as nitrite (NO_2_^−^) or nitrate (NO_3_^−^) (4). Such flexible energy metabolism of this bacterium enables an unrestricted exchange between heterotrophic, mixotrophic, and autotrophic growth conditions. Moreover, the ongoing research into the metabolism of C. necator reveals a wide range of metabolic activities, which can offer unique pathways, intermediates, and products of biotechnological interest (5–8).

Alongside its beneficial use in the process of production of biodegradable plastics (1), C. necator H16 has gained prominence as a chassis for the production of fuels, chemicals, and proteins (9–11), including ethanol, isopropanol, isobutanol, 3-methyl-1-butanol, methyl ketones, alkanes and alkenes, 2-hydroxyisobutyrate, and fatty acids (12–19). Several studies demonstrated that use of C. necator allows achievement of a high cell density, more than 200 g/liter of biomass, without accumulating growth inhibitory organic acids and seemingly precluding formation of inclusion bodies in recombinant protein production (9, 10, 20). These beneficial characteristics imply that C. necator H16 can be used as an alternative expression host.

To develop and optimize biosynthetic pathways in metabolically engineered microorganisms, genome alterations often require either an adjustment of gene expression or an introduction of heterologous genes, in both cases utilizing functional genetic elements that control the gene expression (21). Such genetic elements include promoters (constitutive and inducible) and ribosome binding sites (RBSs), with latter containing Shine-Dalgarno (SD) sequence and in some cases other regulatory mRNA elements, such as palindromic (forming stem-loop structure) or/and A/U-rich sequences (22–26). A few studies have reported on the characterization of functional genetic elements in C. necator H16. The heterologous inducible promoters P_*araBAD*_ (l-arabinose), P_*xylS*_ (*m*-toluic acid), P_*lac*_ (lactose), P_*hpdH*_ (3-hydroxypropionic acid), and P_*rhaBAD*_ (l-rhamnose) (16, 27–29), the synthetic anhydrotetracycline- and cumate-inducible promoters (30, 31), and several native promoters (27) have been shown to be suitable to control and drive gene expression in C. necator. Escherichia coli-derived promoters P_*lacUV5*_, P_*trc*_, and P_*trp*_ have been applied to regulate expression of R-specific enoyl coenzyme A (enoyl-CoA) hydratases, allowing modulation of the proportion of the (*R*)-3-hydroxyhexanoate monomer in poly[(*R*)-3-hydroxybutyrate-co-3-hydroxyhexanoate] (32). A few constitutive promoters, derived from bacteriophage T5, containing only a core promoter sequence displayed much higher activity in C. necator than the commonly used strong promoter P_*tac*_ (20). In addition, Bi and coworkers (16) have demonstrated that inclusion of a 5′ mRNA stem-loop structure upstream of the RBS sequence increases gene expression from inducible promoter P_*araBAD*_ 2-fold in C. necator H16.

A strong need to expand the synthetic biology toolbox remains, aiming to broaden the range of functional genetic elements for controlling gene expression in C. necator. Inducible control systems involving either positive or negative regulators are also required for highly controllable circuits in synthetic biology and biotechnology applications. Importantly, the quantitative and comparative evaluation of existing and new functional genetic elements is needed to facilitate genetic and metabolic engineering. The aim of this study was to design, build, and quantitatively characterize sets of constitutive promoters and RBSs, which would provide a wide range of strengths for gene expression control. Four inducible systems, known to have practical application in other microbial chassis, were assembled and characterized in a comparative manner. Inducible systems and a set of RBSs are applied for controlling expression of the *ispS* gene, leading to variable isoprene production.

## RESULTS

### Assembly and quantitative evaluation of insulated constitutive promoters.

**FIG 1:**
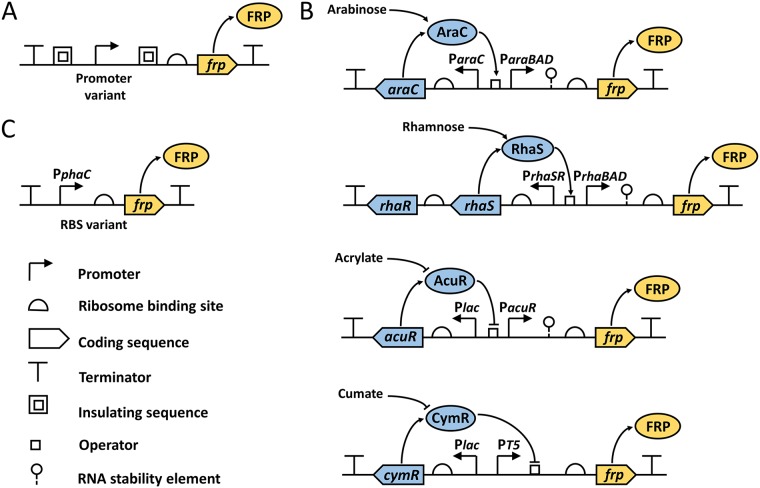
Design of promoter and RBS constructs. Shown are SBOL (67) visual representations of constructs that contain constitutive promoter (A), four inducible systems (B) and RBS (C). SBOL visual icons are specified. FRP, fluorescent reporter protein.

To generate libraries of functional genetic elements, a quick, reliable and preferably one-pot-reaction strategy allowing a simultaneous assembly of multiple DNA fragments is desirable. Taking this into account, for assembly of the promoter library we chose to employ the uracil-specific excision reagent (USER)-based engineering and cloning method (33). Using pBBR1MCS-2 (34) and modular pMTL71101 as plasmid backbones, we constructed vectors pBBR1-USER and pMTL71107, respectively, containing a USER cassette to assist with the assembly (see Materials and Methods for details). In total, 26 promoter variants were constructed in both vectors. To enable measurement of their strengths, promoters were transcriptionally fused to a gene encoding enhanced yellow fluorescence protein (eYFP) and a strong RBS, a 27-nucleotide upstream sequence of the bacteriophage T7 gene *10* (T7*g10*) (35), was placed directly upstream of the reporter gene. A variety of promoter strengths was achieved by combining core promoter sequences of previously characterized promoters from C. necator, E. coli, and bacteriophages T4 and T5 (3, 36–38) with upstream and downstream insulation sequences (Fig. 1A). Such insulation sequences have been reported to increase the predictability of promoters by reducing the influence of the different sequence/genomic context (39). The core promoter sequences used in this study are listed in Table 1.

**TABLE 1:**
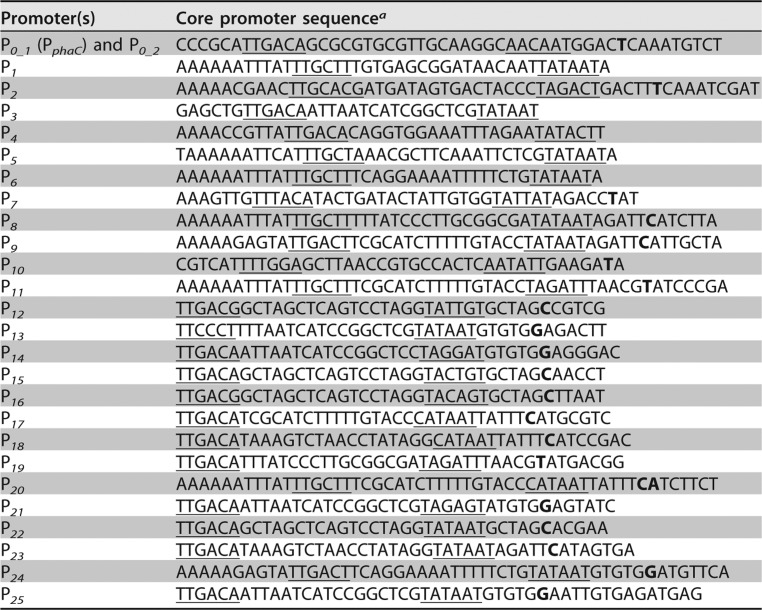
Core promoter sequences used for insulated constitutive promoter library

^*a*^The core promoter sequence containing bacterial RNA polymerase binding determinants, −10 and −35 boxes (underlined), and transcription start site (in bold) was predicted using the Neural Network Promoter Prediction tool (68).

**FIG 2:**
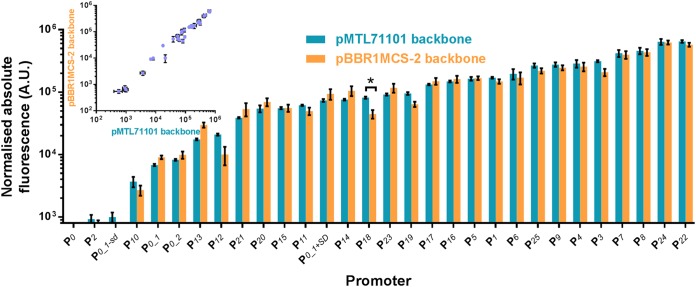
Quantitative evaluation of constitutive promoter strengths. The strengths of different promoters were determined by their abilities to drive expression of the fluorescence reporter. C. necator H16 cells harboring the variants of promoter-reporter constructs in either the pBBR1MCS-2 or pMTL71101 backbone were grown in MM as described in Materials and Methods. To determine normalized absolute fluorescence, the optical density at 600 nm and EYFP fluorescence were measured. Error bars represent standard deviations from three biological replicates. The asterisk indicates statistically significant difference between promoter strengths in different vector backbones (*P* < 0.05, unpaired *t* test). Correlation between strengths of respective promoters placed in two different plasmid backbones, pBBR1MCS-2 and pMTL71101, is shown in the inset.

Plasmids with insulated constitutive promoters were transformed into C. necator H16 by electroporation. A promoterless construct (P_*0*_) was included as negative control. Resulting C. necator strains were cultured in minimal medium (MM) and the single-time-point fluorescence of the logarithmically growing cells was determined as a measure of eYFP protein concentration normalized to the cell density (Fig. 2). A majority of promoters were significantly stronger than the native P_*phaC*_ (P_*0_1+SD*_) or any other C. necator promoters characterized so far, with promoters P_*22*_ and P_*24*_ showing 9-fold higher activity. Strengths of respective promoters were comparable in the pBBR1MCS-2 and pMTL71101 vector backbones (Fig. 2, inset). Activities of the insulated promoters P_*1*_, P_*2*_, P_*4*_, P_*5*_, and P_*6*_, which contained core promoters P_*T5*_, P_*H16_B1772*_, P_*j5*_, P_*h207*_, and P_*n25*_, respectively, correlated with previously published data (20) obtained using enhanced green fluorescent protein (eGFP) as a reporter and corresponding core promoter elements (see Fig. S3 in the supplemental material). Notably, a wide coverage of promoter strengths spanning over a 700-fold range was achieved in the developed promoter library.

### Inducible systems and their quantitative evaluation in C. necator H16.

Inducible promoters are indispensable tools within the context of synthetic biology to control gene expression. To date, a number of transcription-based inducible systems have been tested in C. necator (16, 27–31). However, the genetic contexts in which they have been evaluated differ considerably, making it difficult to compare their outputs.

We chose to evaluate and compare two positively and two negatively regulated inducible systems. The AraC/P_*araBAD*_-l-arabinose-, RhaRS/P_*rhaBAD*_-l-rhamnose-, and CymR/P_*cmt*_-cumate-inducible systems have been employed previously in C. necator (16, 29, 31). The fourth system is regulated by AcuR, a repressor protein from Rhodobacter sphaeroides mediating gene expression from P_*acuRI*_ in the presence of acrylate (40). Reporter systems were designed as described recently for the modular reporter plasmid pEH006. It contains the l-arabinose-inducible system and was demonstrated to be a suitable original vector for the evaluation of inducible systems (28). Vectors were constructed in such a way that the transcriptional regulator (TR) coding sequences are located in opposite direction of the *rfp* reporter gene (Fig. 1B). The genes encoding the activator proteins AraC and RhaRS are expressed from their native promoters to maintain TR-mediated autoregulation (41, 42), whereas the E. coli *lac* promoter including *lacI* operator was used to control transcription of the genes encoding the transcriptional repressors AcuR and CymR. Utilization of repressible *lac* promoter enabled elimination of a toxic effect caused by overexpression of transcriptional repressors in E. coli when assembling vectors. Expression of *rfp* is driven by the corresponding inducible promoter. The native l-arabinose-, l-rhamnose-, and acrylate-inducible promoters were designed to harbor a T7*g10* mRNA stem-loop structure with the aim to enhance gene expression through improved RNA stability (43). For the cumate-inducible system, a synthetic promoter composed of the phage T5 promoter and the operator sequence of the *cmt* operon was employed (44). The same RBS as for the constitutive promoters was used in all of the constructs.

**FIG 3:**
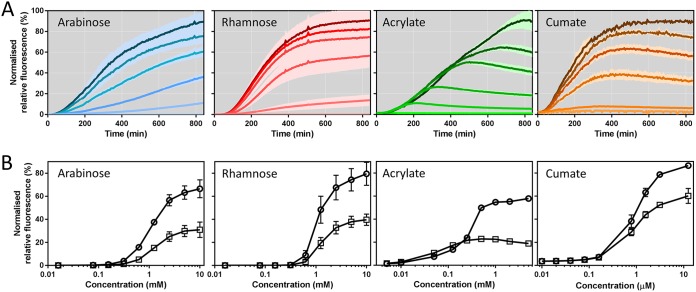
Induction kinetics and dose response for the l-arabinose-, l-rhamnose-, acrylate-, and cumate-inducible systems. (A) Normalized relative fluorescence of C. necator H16 harboring the l-arabinose-, l-rhamnose-, acrylate-, and cumate-inducible systems. Inducers were added at time zero and fluorescence was monitored for 14 h. The darker the color shade, the higher the inducer concentration. l-Arabinose was supplemented to final concentrations of 10, 2.5, 1.25, 0.625, and 0.313 mM or omitted. l-Rhamnose was supplemented to final concentrations of 10, 5, 2.5, 1.25, and 0.625 mM or omitted. Acrylate was supplemented to final concentrations of 5, 1, 0.5, 0.25, and 0.05 mM or omitted. Cumate was supplemented to final concentrations of 12.5, 3.13, 1.56, 0.78, and 0.16 μM or omitted. The standard deviations from three biological replicates are illustrated as lighter shading above and below the induction kinetics curve. (B) Dose response of C. necator H16 harboring the l-arabinose-, l-rhamnose-, acrylate-, and cumate-inducible systems four (square) and eight (circle) hours after inducer addition. Error bars represent SDs from three biological replicates.

Plasmids harboring inducible systems were transformed into C. necator H16 and analyzed for fluorescence output over time at different inducer concentrations (Fig. 3A). The resulting dose-response curves provide information about each system's dynamic range (Fig. 3B). For example, gene expression controlled by the l-arabinose-inducible system can be fine-tuned in the range between 0.313 and 2.5 mM l-arabinose for a linear output. The exponential increase in fluorescence output stretches more widely, between 0.016 and 1.25 mM. Furthermore, absolute normalized fluorescence values were used to calculate the red fluorescent protein (RFP) synthesis rate at any time point during the time course of experiment. The resulting maximum synthesis rate was fitted to the corresponding inducer concentration using a Hill function (Fig. S2), providing key parameters such as the maximum possible rate of RFP synthesis, the Hill coefficient, or the inducer concentration mediating half-maximal RFP synthesis (Table S3). According to the fitted data, the l-rhamnose-inducible system demonstrated the highest induction cooperativity (*h* = 3.88). It requires a minimum concentration of 0.156 mM l-rhamnose to be activated and achieves about 85% of maximum expression at 2.5 mM. The acrylate- and cumate-inducible systems generally require lower inducer levels to initiate gene expression. Moreover, the range of inducer concentration mediating a linear fluorescence output spans more than 1 order of magnitude (5 to 125 μM and 0.08 to 1.56 μM, for acrylate and cumate, respectively) and therefore can be fine-tuned more easily.

Regardless of inducer concentration, we noticed that the absolute fluorescence, corrected by fluorescence that derives from basal promoter activity, generally increased during the time course of experiment. This was not the case for the acrylate-inducible system. Six hours after inducer addition, the increase in absolute fluorescence output facilitated by acrylate concentrations of 1.25 mM and less was at the same level as mediated by basal P_*acuRI*_ activity. This behavior is also reflected in its dose-response curve (Fig. 3B). Normalized fluorescence values for acrylate concentrations of 1.25 mM and less were lower after 8 h of induction than that after 4 h, whereas the increase in the acrylate concentration enabled extended expression of the reporter gene. We hypothesized that this type of transient gene expression can be caused by inducer degradation. To test whether acrylate is catabolized by C. necator H16, a metabolite consumption assay was performed as described in Materials and Methods. As predicted, acrylate was coconsumed simultaneously with the primary carbon source fructose (Fig. S4). Four hours after supplementation with 5 mM acrylate, 49% of the initial amount was consumed. Upon depletion of inducer, gene expression was maintained at the level of basal promoter activity. Neither of the other three inducers, l-arabinose, l-rhamnose, or cumate, appeared to be consumed. C. necator H16 was not able to grow in MM supplemented with these three inducers as the sole carbon source (data not shown).

**FIG 4:**
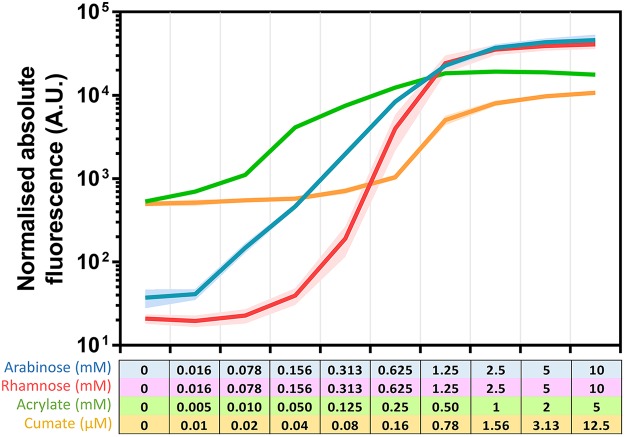
Induction dynamics for the l-arabinose-, l-rhamnose-, acrylate-, and cumate-inducible systems. Shown is normalized absolute fluorescence of C. necator H16 harboring the l-arabinose-, l-rhamnose-, acrylate-, and cumate-inducible systems. Fluorescence was determined for cells in exponential growth phase 6 h after inducer addition. Inducer concentrations are indicated for each system. The standard deviations from three biological replicates are illustrated as lighter shading above and below the dynamics curve.

In addition to their dynamic range, another important characteristic of inducible systems is their induction factor. It was calculated for cells in exponential growth phase, 6 h after the inducer was supplemented. Dividing the maximum normalized fluorescence resulting from the highest inducer concentration by the normalized fluorescence of the uninduced sample yielded induction factors of 1,232, 1,960, 33, and 22 for the l-arabinose-, l-rhamnose-, acrylate-, and cumate-inducible systems, respectively. The induction factors that were achieved by AcuR/P_*acuRI*_ and CymR/P_*cmt*_ were much lower than for the two positively regulated systems due to high background levels of reporter gene expression in the absence of inducer (Fig. 4). The order from the highest to the lowest normalized absolute fluorescence achieved by the four tested systems throughout the time course of experiment for the highest inducer concentration is as follows: l-arabinose > l-rhamnose > acrylate > cumate. The same order applies to the maximum possible RFP synthesis rate, with l-arabinose demonstrating the highest value (Table S3). All of the systems can be considered to activate reporter gene transcription immediately after inducer addition. Fluorescence above background levels could be detected within 30 min, representing the time which is required for RFP production and maturation (45).

To evaluate the influence of the T7*g10* mRNA stem-loop structure sequence on inducible gene expression, it was removed from the plasmid containing the l-arabinose-inducible system. The relationship between fluorescence response and inducer concentration for the l-arabinose-inducible system without the stem-loop structure sequence was similar to the construct harboring the stem-loop (Fig. S5). A linear fluorescence output was achieved by addition of l-arabinose from 0.313 to 2.5 mM. The induction factor was considerably higher (2,900-fold), mainly due to an 8-fold lower background level of RFP synthesis. However, removing the stem-loop also decreased absolute normalized fluorescence levels 3.6-fold at an inducer concentration of 10 mM (Fig. 4; see also Fig. S5).

### Assembly and quantitative evaluation of RBS library.

Similarly to the promoter, an RBS has a large impact on the protein synthesis level and, therefore, can govern compound biosynthesis in engineered microorganisms. To balance quantities of enzymes in a metabolic pathway, use of RBSs with variable strengths can help achieve different levels of protein synthesis from individual genes that are organized in a single operon and transcribed from the same promoter.

**FIG 5:**
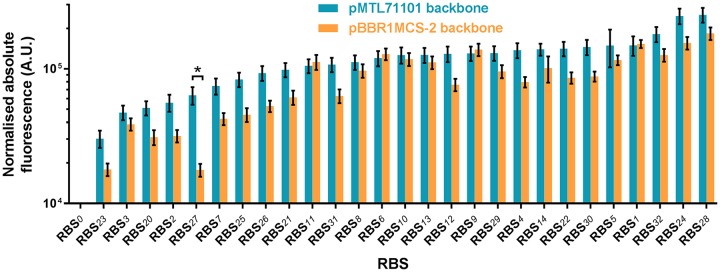
Quantitative evaluation of RBSs. The strengths of different RBSs were evaluated by using the fluorescence reporter. C. necator H16 cells harboring the variants of RBS-reporter constructs in either pBBR1MCS-2 or pMTL71101 backbone were grown in MM as described in Materials and Methods. To determine normalized absolute fluorescence, the optical density at 600 nm and EYFP fluorescence were measured. Error bars represent standard deviations from three biological replicates. The asterisk indicates statistically significant difference between RBS strengths in different vector backbones for (*P* < 0.05, unpaired *t* test).

In this study, we used an RBS calculator (46) to develop a range of RBSs with variable strengths (Table 2). Employing the USER-based method similarly to the constitutive promoter library, 27 RBS sequences were assembled in two alternative vector backbones, pBBR1MCS-2 and pMTL71101, under the control of the *phaC* promoter (Fig. 1C). Resulting constructs were transformed into C. necator H16. The RBS strengths were evaluated by measuring the single time point fluorescence of cells in exponential growth phase (Fig. 5). A majority of RBSs showed similar activities in the two pBBR1MCS-2 and pMTL71101 vector backbones, exhibiting more than a 10-fold dynamic range. Only one RBS variant (RBS_*27*_) showed a significantly (*P* < 0.05) lower fluorescence output in the pBBR1MCS-2 vector backbone than that in pMTL71101.

**TABLE 2:**
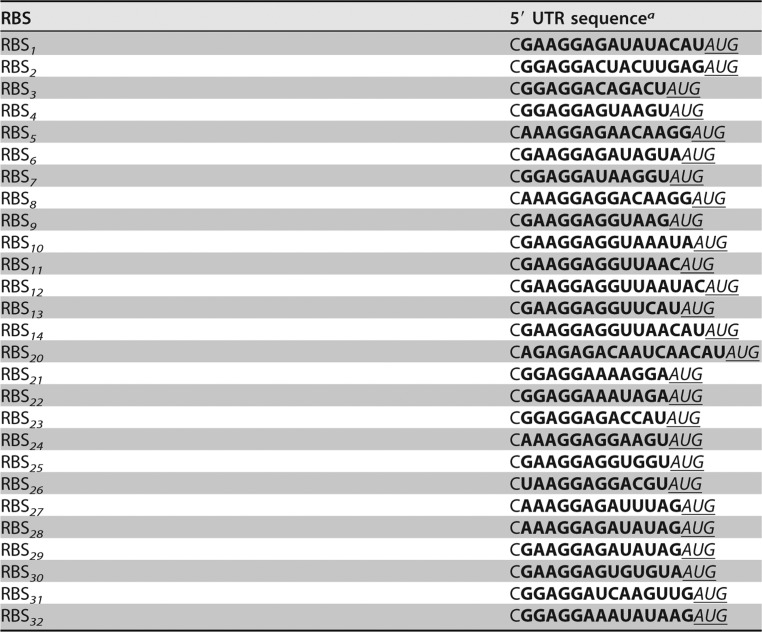
RBSs with variable strengths

^*a*^The unique RBS is in bold, and the gene start codon AUG is in italic and underlined. UTR, untranslated region.

It should be noted that the activity of an RBS can vary extensively depending on the sequence context (21). To assess this, three selected RBSs—weak (RBS_*2*_), medium (RBS_*8*_), and strong (RBS_*1*_)—were evaluated using constructs containing combinations of upstream (promoter) and downstream (reporter) genetic elements, including *eyfp*, driven by the P_*phaC*_, P_*1*_, or P_*rhaBAD*_ promoter, and *rfp*, driven by P_*phaC*_. As expected, the change from medium-strength P_*phaC*_ to the strong P_*1*_ promoter contributed to the overall increase in gene expression but had little effect on the relative strength of the three cognate RBSs, displaying unchanged hierarchy of strengths RBS_*1*_ > RBS_*8*_ > RBS_*2*_ (Fig. S6A). Strikingly, the downstream change of genetic element (*rfp* gene) had a more pronounced effect on the relative strength of RBSs, significantly increasing it for RBS_*1*_ and RBS_*8*_ but not for RBS_*2*_ (Fig. S6B). These findings suggest that variable-strength RBSs can aid in achieving differential levels of protein synthesis. However, depending on the genetic context, the absolute levels of gene expression should be fine-tuned by testing a range of RBS alternatives.

### mRNA stem-loop structure and A/U-rich sequence.

**FIG 6:**
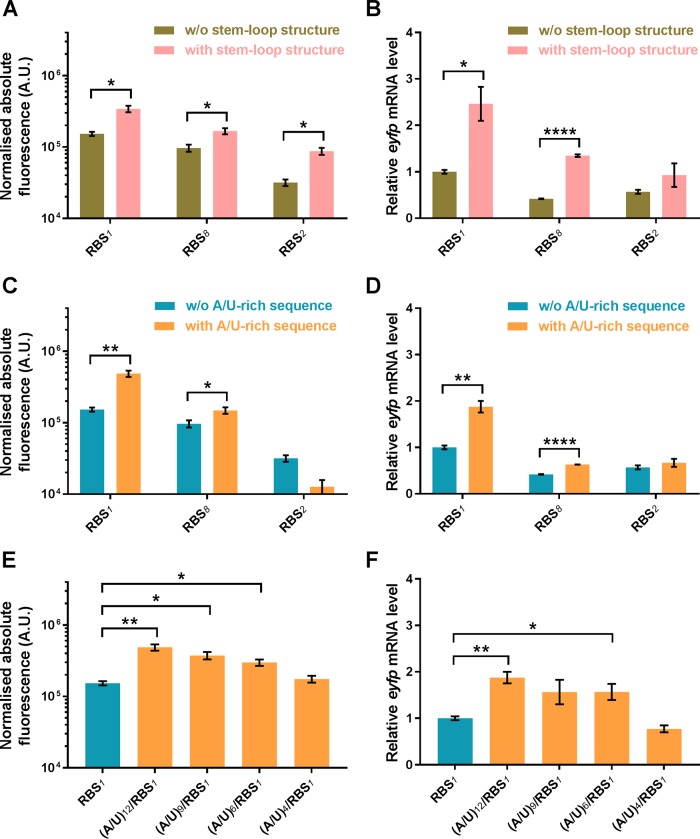
Effect of upstream mRNA stem-loop structure and A/U-rich sequence on translational activity and mRNA abundance. The translational activities of three different RBSs (RBS_*1*_, RBS_*2*_, and RBS_*8*_) with and without upstream T7*g10* mRNA stem-loop structure sequence (A), with and without upstream A/U-rich sequence (C), and with upstream A/U-rich sequences of different lengths (E) were evaluated by using the EYFP fluorescence reporter. To determine normalized absolute fluorescence, the optical density at 600 nm and EYFP fluorescence were measured. The mRNA abundance from constructs with and without upstream T7*g10* mRNA stem-loop structure sequence (B), with and without upstream A/U-rich sequence (D), and with upstream A/U-rich sequences of different lengths (F) were measured by RT-PCR as described in Materials and Methods. Error bars represent standard deviations from three biological replicates. Asterisks indicate statistically significant increase in translational activity or *eyfp* mRNA abundance in the presence of stem-loop structure (A and B) or A/U-rich (C to F) sequences as follows: *, *P* < 0.01; **, *P* < 0.001; and ****, *P* < 0.00001 (unpaired *t* test).

An mRNA stem-loop structure and A/U-rich sequence have been reported previously to increase mRNA stability and/or translation efficiency, contributing to improved gene expression in E. coli (22, 23, 43). To test this in C. necator, the T7*g10* mRNA stem-loop structure (43) and different-length A/U-rich sequences were inserted upstream to the SD sequence of the RBS. The introduction of the stem-loop structure increased fluorescent reporter protein synthesis 1.5- to 3-fold for constructs with all three RBSs (Fig. 6A), whereas the A/U-rich sequence exhibited a significant increase only in case of the construct with RBS_*1*_ (Fig. 6C). The stepwise lengthening of an A/U-rich sequence from 4 to 12 nucleotides resulted in a linear increase of reporter protein synthesis (Fig. 6E). Moreover, the introduction of either mRNA stem-loop structure or A/U-rich sequence contributed to mRNA stabilization (Fig. 6B, D, and F). Overall, these data suggest that depending on the RBS sequence context, A/U-rich sequences can be applied as genetic elements for altering and fine-tuning gene expression, whereas the mRNA stem-loop structure can be used as a universal gene expression enhancer in C. necator H16.

### Control of isoprene biosynthesis using inducible promoters and RBS variants.

Gene expression can result in different output patterns for reporter molecules and metabolites. To evaluate how the variation in gene expression can affect product biosynthesis, a simple, one-enzymatic-reaction pathway extension based on a single gene addition was chosen as a suitable model for investigation. Among several potential targets particularly relevant to the industrial application, production of isoprene using isoprene synthase (IspS) emerged as the most significant. IspS has been demonstrated previously to catalyze production of isoprene from dimethylallyl pyrophosphate (DMAPP) (47, 48), which in C. necator can be produced via the 2-C-methyl-d-erythritol 4-phosphate/1-deoxy-d-xylulose 5-phosphate (MEP/DOXP) pathway. Notably, due to the poor catalytic properties of the enzyme (high *K_m_* and low *k*_cat_), isoprene synthase is considered one of key bottlenecks in isoprene biosynthesis (49).

**FIG 7:**
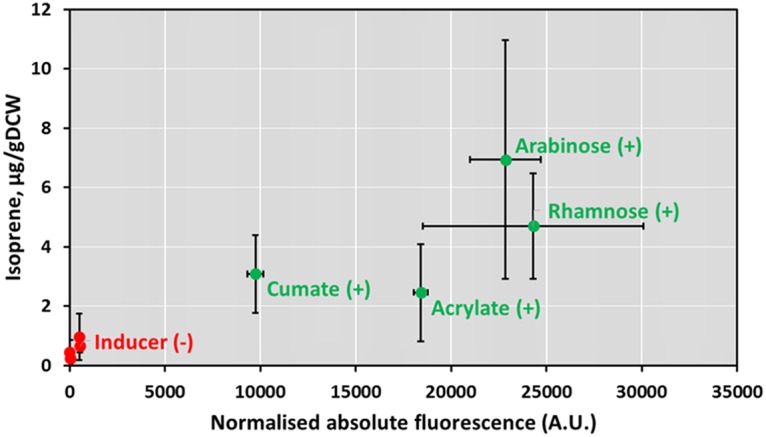
Correlation between gene expression levels and isoprene yields using l-rhamnose-, l-arabinose-, acrylate-, and cumate-inducible systems. Normalized absolute fluorescence of C. necator H16 strains carrying l-rhamnose (RhaRS/P_*rhaBAD*_)-, l-arabinose (AraC/P_*araBAD*_)-, acrylate (AcuR/P_*acuRI*_)-, and cumate (CymR/P_*cmt*_)-inducible systems is plotted against isoprene yield, resulting from strains carrying the *ispS* gene under the control of corresponding inducible systems, in the presence [(+), green dots] or absence [(−), red dots] of inducers. The plotted values represent the normalized fluorescence 6 h and isoprene yield 18 h after induction with 1.25 mM l-rhamnose, 1.25 mM l-arabinose, 0.5 mM acrylate, or 3.13 μM cumate. Error bars represent standard deviations from three biological replicates.

First, to establish whether production of isoprene can be achieved in C. necator H16, and to investigate how gene expression of the enzyme (isoprene synthase) translates into the product of enzymatic reaction (isoprene), *Populus alba ispS* under the control of two positively (AraC/P_*araBAD*_ and RhaRS/P_*rhaBAD*_) and two negatively (AcuR/P_*acuRI*_ and CymR/P_*cmt*_) regulated inducible systems was introduced into C. necator on a plasmid. Induction of *ispS* expression with l-arabinose, l-rhamnose, acrylate, or cumate confirmed that isoprene was biosynthesized to different levels, resulting in a yield up to 7 μg/g of cells (dry weight) (Fig. 7). Moreover, the isoprene yield showed a moderate positive linear correlation with gene expression (measured as fluorescence output) of corresponding inducible systems (*r* = 0.625; only data of induced samples were used in the analysis).

**FIG 8:**
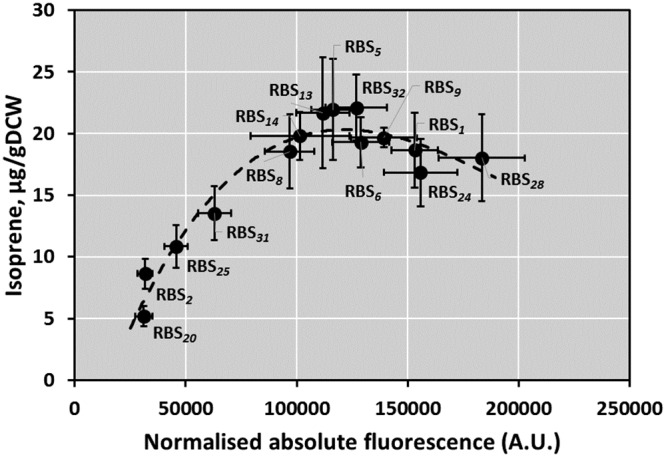
Impact of increase in the strength of RBS on isoprene production. Normalized absolute fluorescence of C. necator H16 strains carrying set of plasmids (pBBR1MCS-2-RBS_*x*_-ispS) with *ispS* gene under the control of the *phaC* promoter and different RBSs is plotted against isoprene yield. Error bars represent standard deviations from three biological replicates.

Second, to evaluate how the designed RBSs can be applied for fine-tuning of gene expression in biotechnology applications and how metabolite production correlates with the translational efficiency, *ispS* was cloned under the control of the P_*phaC*_ promoter and different RBS variants. Isoprene production was measured after 24 h of plasmid-transformed C. necator cell growth as described in Materials and Methods. By applying a range of RBS variants to control translation, we were able to explore the relationship between IspS abundance and isoprene production efficiency (Fig. 8). The data revealed that the highest isoprene yield, 22 μg/g of cells (dry weight), was achieved using medium-strength RBSs, whereas an additional increase in the level of *ispS* expression did not contribute to the further improvement of isoprene production and could potentially have an adverse effect.

## DISCUSSION

Recently, the use of C. necator as a metabolic engineering chassis and biosynthesis host has received renewed interest from academic and industry researchers for its potential to produce proteins, chemicals, and fuels (11, 17, 50). Ability to fix CO_2_ and opportunity to redirect carbon flux from PHB to alternative chemicals open opportunities for use of C. necator in biotechnology applications. However, for production of biotechnology-relevant compounds, the engineering of C. necator is often required, involving genome alterations either by adjusting gene expression or by introducing heterologous genes, in both cases utilizing functional genetic elements that contribute to gene expression control.

The application of functional genetic elements for controlling gene expression has proven useful in metabolic engineering, particularly when building biosynthetic pathways and fine-tuning cellular metabolism. At the same time, achieving distinctive steady-state levels of gene product has been shown to play a critical role for optimal function of the pathway or whole metabolic network (51). Steady-state levels of gene expression can be controlled by using promoters of different strengths, for regulating transcriptional levels, and RBSs, for managing translational efficiency (52). Notably, a substantial amount of research has been dedicated to characterization of promoter and RBS variants in biotechnology-relevant bacterial chassis (21, 26, 53–56).

Building on previous studies with E. coli and bacteriophages (36–38), we quantitatively evaluated a set of variable-strength constitutive promoters composed of −35 and −10 sequences with upstream and downstream insulation. By using the latter, the influence of genomic context can be reduced, as demonstrated previously (39). This concept was supported by a high level of correlation between experimental data of this study and previously reported strengths for a small set of promoters (20). Within our library, promoters were identified with strength spanning a >700-fold dynamic range. At least four promoter variants possessed a higher activity than the strongest promoter currently characterized for C. necator H16, P_*j5*_ (20).

Several heterologous inducible promoters, including l-arabinose, *m*-toluic acid, lactose, 3-hydroxypropionic acid, and l-rhamnose, have been individually characterized previously (16, 27–29). In this study, we have taken a more systematic approach, by evaluating two positively (l-arabinose and l-rhamnose) and two negatively (acrylate and cumate) regulated inducible systems using standardized experimental conditions. In our experimental design and using MM, the positively regulated inducible systems exhibited a tighter control of gene expression than the negatively regulated ones. The highest induction level was achieved using the l-arabinose-inducible system, whereas the l-rhamnose-inducible system exhibited the highest induction factor. The acrylate- and cumate-inducible systems showed significantly lower induction and higher background levels. The application of the acrylate-inducible system in C. necator is limited due to consumption of acrylate by this bacterium. l-Arabinose-, l-rhamnose-, and cumate-inducible systems are suitable for continuous activation of gene expression as well as biosensors. Notably, the cumate-inducible system appeared to be most sensitive, responding to the nanomolar to micromolar range of inducer concentrations. Overall, the systems' dose-response curves are consistent with data from previous induction experiments with C. necator and E. coli (29, 31, 57). However, even though the induction factor of the l-arabinose-inducible system corresponded to the one obtained using sodium gluconate as a carbon source (28), induction factors of the two positively regulated systems were higher than in other studies published so far (16, 27, 29). This variation exemplifies the importance of standardized experimental conditions and comparative studies.

The mRNA secondary structure as well as nucleotide sequence composition, such as the A/U-rich sequence, can also contribute significantly to gene expression regulation. The introduction of A/U-rich sequences into the RBS, upstream of the SD sequence, increases translational efficiency and the stability of mRNA in E. coli, which is thought to result from improved 30S ribosomal subunit binding to the RBS sequence, accelerating recruitment of the ribosome (22). On the other hand, A/U-rich regions have been shown to be a target for RNase E that initiates a decay of mRNA (58). These observations can be explained by two alternative rationales that are, however, not mutually exclusive: (i) the A/U-rich sequence reduces the probability of forming mRNA secondary structures in the translation initiation region, facilitating ribosome binding, which, in turn, hinders both RNase E access to and cleavage of A/U-rich sequence, and (ii) 30S ribosomal protein S1, contributing to the 30S/mRNA interaction, protects from RNase E cleavage and accelerates recruitment of the ribosome. In C. necator H16, we observed that the inclusion of an A/U-rich sequence in the RBS region has a positive effect on gene expression, contributing to increased translational efficiency and the stability of mRNA. Notably, C. necator possesses both the gene *rpsA* (locus tag H16_A0798), encoding protein S1 with 67% identity (94% coverage) to the E. coli K-12 homologue, and the genes *cafA1* and *cafA2* (locus tags H16_A0909 and H16_A2580), encoding potential RNase E/G superfamily members with 38% identity (42% coverage) and 52% identity (67% coverage), respectively, to the E. coli K-12 homologues. These results underline existing similarities of gene expression control between members of gammaproteobacteria (E. coli) and betaproteobacteria (C. necator).

The application of inducible systems and selection of RBSs to control isoprene production revealed that it is important to fine-tune the gene expression to the specific level in order to achieve the optimal yield of isoprene. C. necator H16 cells transformed with plasmids harboring the *ispS* gene under the control of medium-strength RBS yielded the highest isoprene production, while further increases in gene expression resulted in no further increase and even decreases in levels of the product. This was somewhat surprising, since the gene expression data using fluorescence reporter showed that the increase in strength of RBS translated to higher yields of protein. The observed limitation in the isoprene production can be explained by several lines of reasoning: (i) if the overproduction of isoprene synthase is toxic, cells can potentially reduce this effect by either turning down expression or increasing degradation of IspS protein; (ii) due to the inherently high *K_m_* of IspS, the reduced availability of substrate can become a limiting factor in the biosynthesis of isoprene (49, 59); (iii) since isoprene synthase competes with geranyl pyrophosphate synthase and farnesyl pyrophosphate synthase for DMAPP, the overexpression of IspS can cause a depletion of prenyl phosphates (e.g., undecaprenyl phosphate) required for biosynthesis of various cell wall polymers (60); (iv) as it has been previously reported that isoprene synthase forms inclusion bodies when overexpressed (49), protein aggregation can inhibit the enzymatic activity of IspS.

To conclude, the previous work has delivered a limited toolbox of functional genetic elements suitable for application in the metabolic engineering of C. necator. In this study, we have quantitatively evaluated and compared an array of new genetic elements, including constitutive and inducible promoters, RBSs, mRNA stem-loop structure, and A/U-rich sequences, expanding the choice of tools. Our quantitative results will inform design and engineering of synthetic pathways and genetic circuitry in C. necator H16 and other related bacterial species.

## MATERIALS AND METHODS

### Bacterial strains.

C. necator H16 (DSMZ-428, ATCC 17669) was purchased from DSMZ (Braunschweig, Germany) and used in all experiments, including fluorescence assays, RNA isolation, and isoprene production, as described in the following sections. E. coli TOP10 (Invitrogen, Carlsbad, CA) was used for cloning and plasmid propagation.

### Oligonucleotides, chemicals, and enzymes.

Oligonucleotide primers were synthesized by Eurofins Genomics (Ebersberg, Germany) and are listed in Table S1 in the supplemental material. l-(+)-Arabinose (99% purity; Acros Organics, Thermo Fisher Scientific, USA; catalogue no. 365181000), l-rhamnose monohydrate (≥99% purity; Sigma-Aldrich, USA; catalogue no. R3875), magnesium acrylate (95% purity; Alfa Aesar, Thermo Fisher Scientific, USA; catalogue no. 42002; lot no. L14619), 4-isopropylbenzoic acid (cumic acid; ≥98% purity; Acros Organics, Thermo Fisher Scientific, USA; catalogue no. 412800050), and d-(−)-fructose (≥99% purity; Sigma-Aldrich, USA; catalogue no. F0127) were used as inducers for assaying inducible systems or substrates in metabolite consumption assays. Isoprene (99% purity; Alfa Aesar, Thermo Fisher Scientific, USA; catalogue no. L14619) was used as a standard for isoprene yield quantification. Restriction enzymes were purchased from New England BioLabs (USA).

### Plasmid construction. (i) Insulated constitutive promoter and RBS libraries.

The core promoter sequences used to construct the library of insulated constitutive promoters are listed in Table 1. The following is an example of insulated promoter sequence: promoter P_*15*_, GCTGAGGGAAAGTACCCAAAAATTCATCCTTCTCGCCTATGCTCTGGGGCCTCGGCAGATGCGAGCGCTGCATACCGTCCGGTAGGTCGGGAAGCGTGCAGTGCCGAGGCGGATTAATCGATttgacagctagctcagtcctaggtactgtgctag**c**aacctGAATTCACTAGTTTAACTTTAAGAA. The insulations preceding (122 bases) and following (25 bases) the core promoter are both shown in uppercase and are partially derived from the upstream region of the *phaC* promoter and upstream nucleotide sequence of the T7 gene *10* RBS, respectively. For each insulated promoter, only the core sequence (in lowercase), including the −35 and −10 hexamers (underlined), up to 12 bases upstream of the −35 box, the 17-base spacer between the −35 and −10 boxes, and up to 20 bases downstream of the −10 box, vary between library members (Table 1). The expected transcription start site is in bold.

Plasmids with insulated constitutive promoters and RBS variants (Table 2) were constructed using the uracil-specific excision reagent (USER) method as described previously (33). In order to generate the USER-compatible vector, a DNA cassette containing *lacZ*α (for blue-white color screening of colonies) flanked by inversely oriented nicking endonuclease Nt.BbvCI sites and restriction endonuclease XbaI sites was synthesized by PCR using oligonucleotide primers P001 and P002 and pGEM-T plasmid (Promega) as a DNA template. To exclude any possible transcriptional readthrough, *rrnB* terminator T2 (5′-AAATTAAGCAGAAGGCCATCCTGACGGATGGCCTTTTTGCGTTT-3′) and truncated version of terminator T1 (5′-ATCAAATAAAACGAAAGGCCTTCGGGCCTTTCGTTTTATCTGTTGTTT-3′) (61) were inserted upstream and downstream, respectively, of the cassette flanked by Nt.BbvCI sites. Within the cassette, each XbaI site was separated from the adjacent Nt.BbvCI site by unique, 5-nucleotide-long spacer sequences, GAAAG and TGTCT. The PCR fragment containing the USER cassette was digested with KpnI and AgeI and cloned into pBBR1MCS-2-PphaC-eyfp-c1 (62). The resulting plasmid, pBBR1MCS-2-USER, was used to excise the KpnI-NheI DNA fragment including the USER cassette. This DNA fragment was also subcloned into modular vector pMTL71101 (see Fig. S1 and Materials and Methods in the supplemental material) through KpnI and NheI restriction sites, yielding plasmid pMTL71107.

All insulated constitutive promoters and RBSs were assembled in vectors pBBR1MCS-2-USER and pMTL71107 by employing the USER method. For this purpose, two overlapping DNA fragments, including promoter, RBS and *eyfp* gene sequences, were generated by PCR with two pairs of oligonucleotide primers, P003 with P005_pX_r (or P007_rbsX_r; “p” and “rbs” represent promoter and RBS, respectively, while “X” is the corresponding number of the promoter or RBS) and P006_pX_f (or P008_rbsX_f) with P004 using plasmid pBBR1MCS-2-PphaC-eyfp-c1 as a template. The overlapping primers, P005_pX_r (or P007_rbsX_r) and P006_pX_f (or P008_rbsX_f), were designed in such a way so that the desired promoter (or RBS sequence) is integrated into the primer sequence. The resulting plasmids with promoter and RBS variants are referred to as pBBR1MCS-2-P_*0*_ to pBBR1MCS-2-P_*25*_ and pBBR1MCS-2-RBS_*0*_ to pBBR1MCS-2-RBS_*32*_ in the pBBR1MCS-2 vector backbone and as pMTL71107-P_*0*_ to pMTL71107-P_*25*_ and pMTL71107-RBS_*0*_ to pMTL71107-RBS_*32*_ in the pMTL71107 vector backbone.

For use as negative controls, the promoterless construct P_*0*_ and SD sequence-free construct RBS_*0*_ (including the complement SD sequence) were generated. P_*0*_ was assembled from the DNA fragment that was PCR amplified using oligonucleotide primers P006_p0_f and P004 from pBBR1MCS-2-PphaC-eyfp-c1 and was cloned into either the pBBR1MCS-2-USER or pMTL71107 vector. The assembly of RBS_*0*_ involved two overlapping DNA fragments generated by PCR with two pairs of primers, P003-P007_rbs0_r and P008_rbs0_f-P004, using same template as described above.

### (ii) mRNA stem-loop and A/U-rich sequence variants.

To construct plasmids pBBR1MCS-2-SL/RBS_*1*_, pBBR1MCS-2-SL/RBS_*2*_, and pBBR1MCS-2-SL/RBS_*8*_, containing RBS variants with upstream T7*g10* mRNA stem-loop (43) (SL), primer P007_SL/rbs_r overlapping with primers P008_SL/rbs1_f, P008_SL/rbs2_f, and P008_SL/rbs8_f were used, respectively. The plasmids pBBR1MCS-2-(A/U)_12_/RBS_*1*_, pBBR1MCS-2-(A/U)_9_/RBS_*1*_, pBBR1MCS-2-(A/U)_6_/RBS_*1*_, and pBBR1MCS-2-(A/U)_4_/RBS_*1*_, bearing RBS variants with A/U-rich (A/U) sequences, were generated using corresponding primers P007_(A/U)_12_/rbs1_r, P007_(A/U)_9_/rbs1_r, P007_(A/U)_6_/rbs1_r and P007_(A/U)_4_/rbs1_r, which all overlapped with primer P008_rbs1_f. The use of primers P007_(A/U)_12_/rbs2_r and P007_(A/U)_12_/rbs8_r overlapping with primers P008_rbs2_f and P008_rbs8_f allowed construction of plasmids pBBR1MCS-2-(A/U)_12_/RBS_*2*_, and pBBR1MCS-2-(A/U)_12_/RBS_*8*_, respectively.

### (iii) Inducible systems.

The inducible systems were cloned into the *C. necator-E. coli* shuttle vector pEH006 (28), based on the modular vector system which has been developed previously (63). All constructed plasmids contained following functional modules: (i) a broad-host-range pBBR1 origin of replication (34), (ii) antibiotic (chloramphenicol or kanamycin) resistance gene, and (iii) promoter/RBS-reporter gene (*eyfp* or *rfp*) fusion. The utilization of restriction sites for rare-cutter enzymes SbfI, PmeI, FseI, and AscI made these functional modules interchangeable (63) for pMTL71107- and pEH006-derived plasmids.

The l-rhamnose-inducible system RhaRS/P_*rhaBAD*_ was amplified with oligonucleotide primers EH007_f and EH008_r from pJOE7784.1 (64) and cloned into pEH006 by AatII and XbaI restriction sites, resulting in pEH002.

pEH005 served as the backbone for assembly of negatively regulated systems. It is the same vector as pEH006 except for two elements. Instead of an *rrnb* terminator, T1, downstream of the inducible system's transcriptional regulator, it contains an *rrnb* terminator, T2. Furthermore, the l-arabinose-inducible system was replaced by P_*lac*_-*tetR* and P_*tetA*_. It was assembled by using the NEBuilder Hifi DNA assembly method. Oligonucleotide primers EH025_f and EH026_r, EH027_f and EH028_r were used to amplify the vector backbone and *tetR* from pEH006 and pJOE7801.1 (64), respectively. Primer overhangs were designed to contain *rrnb* terminator T2 and a PvuI restriction site to be able to replace the incorporated *lac* promoter.

The vector containing the acrylate-inducible system, pEH020, was assembled by using the NEBuilder Hifi DNA assembly method. Oligonucleotide primers EH048_f and EH057_r and primers EH056_f and EH055_r were used to amplify the vector backbone for negatively regulated systems and the acrylate inducible system (40) from pEH005 and Rhodobacter sphaeroides 2.4.1 genomic DNA, respectively.

The vector containing the cumate-inducible system, pEH040, was assembled by using the NEBuilder Hifi DNA assembly method. Oligonucleotide primers EH048_f and EH012_r, EH011_f and EH109_r, EH112_f and EH111_r, and EH114_r and EH113_f were used to amplify the vector backbone for negatively regulated systems, the transcriptional regulator *cymR*, and the cumate-inducible promoter P_*cmt*_ from pEH005 and pNEW (44), respectively.

The l-arabinose-inducible system without the T7*g10* stem-loop structure was amplified with oligonucleotide primers EH310_f and EH016_r from E. coli MG1655 genomic DNA and cloned into pEH006 by AatII and NdeI restriction sites, resulting in pEH176.

### (iv) Plasmids for isoprene production.

Plasmids harboring codon-optimized Populus alba isoprene synthase gene (*ispS*; sequence can be found in Materials and Methods in the supplemental material) under the control of either different RBS variants or inducible systems were used for isoprene production. To construct pBBR1MCS-2-RBS_*x*_-ispS series with RBS variants (“*x*” is the corresponding number of RBSs), *eyfp* was replaced with *ispS* gene through restriction sites NdeI and BamHI or by the USER method. To construct plasmids for isoprene production using l-rhamnose-, l-arabinose-, acrylate-, and cumate-inducible systems, *rfp* was replaced with *ispS* through restriction sites NdeI and BamHI for plasmids pEH002, pEH006, and pEH020 and through NdeI and AflII for plasmid pEH040, resulting in pEH002-ispS, pEH006-ispS, pEH020-ispS, and pEH040-ispS, respectively. The DNA fragment containing *ispS* was prepared by either restriction digestion of plasmid pBBR1MCS-2-RBS_*1*_-ispS with NdeI and BamHI or by PCR amplification using plasmid pBBR1MCS-2-RBS_*1*_-ispS as a template and oligonucleotide primers P009_ispS_f and P010_ispS_r, followed by restriction digestion with NdeI and AflII.

All plasmids constructed in this work were verified by Sanger sequencing and listed in Table S2.

### Fluorescence measurements.

For single time point fluorescence measurements, plasmid-transformed C. necator H16 cells were grown in minimal medium (MM) (65) with 4 mg/ml of fructose and 300 μg/ml of kanamycin at 30°C. Cells forming fresh single colonies were inoculated into 2 ml of medium and grown overnight. Ten microliters of overnight culture was added to 390 μl of medium in a 96-deep-well plate (2.0 ml, round wells with round bottoms; STARLAB International GmbH, Germany; catalogue no. E2896-2110) and grown for 24 h. Subsequently, 20 μl of cell culture was subcultured into 380 μl of fresh medium and grown for up to 24 h in a 96-deep-well plate. A total of 150 μl of cell culture in exponential growth phase was transferred into a 96-well microtiter plate (flat and clear bottom, black; Greiner One International, Germany; catalogue no. 655090). The fluorescence was measured in an Infinite M1000 PRO (Tecan, Switzerland) plate reader by using a fluorescence bottom reading mode. For eYFP fluorescence measurement, an excitation wavelength of 495 nm with a 10-nm bandwidth, an emission wavelength of 530 nm with a 10-nm bandwidth, and gain were manually set to 65%. To normalize fluorescence by optical cell density, absorbance was measured using wavelength of 600 nm with a 5-nm bandwidth at the same time. RFP fluorescence was measured using 585 nm as the excitation wavelength and 620 nm as the emission wavelength. The gain factor was set manually to 80%. Time course fluorescence measurements were performed in MM with 4 mg/ml of fructose as the carbon source as described previously (28).

### Normalized relative fluorescence and fluorescent protein synthesis rate.

Relative normalized fluorescence values were calculated to display the fluorescence output of the four evaluated inducible systems on a common axis. This was performed by dividing absolute normalized fluorescence values by 110% of the highest value achieved during the time course of experiment.

The normalized absolute fluorescence values were used to calculate the RFP synthesis rate for each inducer concentration at any time as reported previously (57). The resulting maximum synthesis rate was fit to the corresponding inducer concentration using the Hill function:

(1) $${\left(\frac{\Delta \text{RFP}}{\Delta t}\right)}_{\text{max}}=v_{\text{max}}\times \frac{I^{h}}{I^{h}+K_{m}^{h}}+v_{\text{min}}$$

The parameters correspond to the time (*t*), the maximum RFP synthesis rate (*v*_max_), the inducer concentration (*I*), the Hill coefficient (*h*), the inducer concentration that results in half-maximal RFP synthesis (*K_m_*), and the basal rate of RFP synthesis (*v*_min_). The fitted data for each system are illustrated in Fig. S2. The resulting parameters are summarized in Table S3.

### RNA preparation and real-time PCR.

The wild-type and plasmid-transformed C. necator H16 strains were grown in MM containing 4 mg/ml of fructose and 300 μg/ml of kanamycin. The cells were subcultured twice and grown aerobically for up to 24 h each time in 50-ml conical centrifuge tubes with orbital shaking at 30°C. Subsequently, the culture volume equivalent to an optical density at 600 nm (OD_600_) of 1 for cells in exponential growth phase was pelleted by centrifugation at 13,000 × *g* for 1 min. The pellet was resuspended in 1 ml of TRI reagent (Sigma) and stored at −80°C. RNA was extracted using TRI reagent (Sigma) according to the manufacturer's recommendations. The samples were treated with 2 units of RQ1 RNase-free DNase (Promega), and 2 μg of total RNA was used for cDNA synthesis in a 20-μl reaction volume using the ProtoScript II first-strand cDNA synthesis kit (New England BioLabs). A real-time PCR analysis was performed in a 20-μl reaction volume using LuminoCt SYBR green qPCR ReadyMix (Sigma) and a Light Cycler 480 II (Roche). The relative quantification of mRNA levels was performed using the threshold cycle (ΔΔ*C_T_*) method (66). All biological samples were assayed in duplicates. Primers used for RT-PCR were P011_16S_f and P012_16S_r (for 16S RNA) and P013_yfp_f and P014_yfp_r (for the *eyfp* gene).

### Isoprene production.

Freshly grown overnight cultures of plasmid-transformed C. necator H16 strains were inoculated to an OD_600_ of 0.1 into 10 ml of MM containing 4 mg/ml of fructose and 50 μg/ml of chloramphenicol and incubated at 30°C with vigorous shaking in sealed 60-ml serum bottles. For constructs with the *ispS* gene under the control of inducible promoters, the medium was supplemented with either 1.25 mM l-rhamnose, 1.25 mM l-arabinose, 0.5 mM acrylate, or 3.13 μM cumate. Gas samples from the headspace were taken for isoprene analysis 18 h after induction. Cultures of C. necator H16 strains containing plasmids with different RBS variants and *ispS* under the control of the constitutive promoter P_*phaC*_ were sampled after 24 h of growth in sealed 60-ml serum bottles.

### Analytical methods.

The cell absorbance was measured in a 1-cm-path-length cuvette using BioMate 3S UV-visible (UV-Vis) spectrophotometer at 600 nm (Thermo Fisher Scientific, USA).

For isoprene quantification, gas samples were collected from the headspace of sealed 60-ml serum bottles containing 10 ml of culture. Isoprene was detected by gas chromatography (GC) using the instrument Focus GC (Thermo Fisher Scientific, USA) equipped with a flame ionization detector and an HP-AL/S column (30-m length, 0.25-mm diameter; Agilent Technologies). Nitrogen gas was used as the carrier gas at a flow rate of 2 ml/min. The injector, oven, and detector temperatures were maintained at 220°C, 120°C, and 250°C, respectively. The injection volume was 1 ml. The yields of isoprene per gram of cells (dry weight) were estimated from standard curves generated by analyzing known quantities of isoprene. Dry weight was determined by washing cells from a 20-ml culture in distilled water and separation by centrifugation, followed by vacuum-freeze-drying and weighing cell pellet with microbalance.

The metabolite consumption assay was performed as described previously (28). Cells were inoculated to an OD_600_ of 0.5 in MM supplemented with 20 mM fructose and 5 mM acrylate as carbon sources and allowed to grow for 48 h. Fructose and acrylate concentrations were measured by taking supernatant samples at 0, 2, 4, 8, 24, and 48 h and subjecting them to high-performance liquid chromatography (HPLC) in combination with UV spectroscopy using a Thermo Scientific Ultimate 3000 HPLC system equipped with a Phenomenex Rezex ROA-organic acid H+ (8%) column and a diode array detector with the wavelength set at 210 nm. The concentrations of metabolites in the supernatant were estimated from standard curves generated by analyzing known concentrations of magnesium acrylate and D-(−)-fructose.

### Statistical analysis.

The unpaired *t* test was applied to identify statistically significant differences in the normalized absolute fluorescence and relative mRNA levels. The correction for multiple comparisons was performed using Holm-Sidak method, and alpha level was set at 0.05. The Pearson correlation coefficient *r* was calculated to establish the degree of linear correlation between two variables (e.g., normalized absolute fluorescence and isoprene yield). The *t* test and correlation analysis were performed using GraphPad Prism 7.01 built-in equations.

## Supplementary Material

Supplemental file 1
