# Supplementary material for: Functional Genetic Elements for Controlling Gene Expression in Cupriavidus necator H16
Source: Appl Environ Microbiol. 2018 Sep 17;84(19):e00878-18. doi: 10.1128/AEM.00878-18 (PMC6146998; doi:10.1128/AEM.00878-18)
Supplement: Supplemental file 1 [file zam019188767s1.pdf]

# Supplementary material

Functional genetic elements for controlling gene expression in *Cupriavidus necator* H16

Swathi Alagesan, Erik K. R. Hanko, Naglis Malys*, Muhammad Ehsaan, Klaus Winzer, Nigel P. Minton*

BBSRC/EPSRC Synthetic Biology Research Centre (SBRC), School of Life Sciences, Centre for Biomolecular Sciences, The University of Nottingham, Nottingham NG7 2RD, United Kingdom

*Authors to whom correspondence should be addressed. E-mail: naglis.malys@nottingham.ac.uk; nigel.minton@nottingham.ac.uk

The authors wish it to be known that the first three authors, Swathi Alagesan, Erik K. R. Hanko and Naglis Malys, should be regarded as joint First Authors

# SUPPLEMENTARY MATERIAL AND METHODS

## Construction of modular vector pMTL71101

Vector pMTL71101 was built on the basis of plasmid pMTL83141, which is formed of four modules flanked by 8 bp recognition sites of the type II restriction endonucleases AscI, FseI, PmeI and SbfI (1). Three modifications using plasmid pMTL83141 were carried out as following. First, the 892 bp PmeI-SbfI-flanked DNA fragment encoding the *E. coli* Gram negative replicon ColEI RNA II was replaced with a fragment containing broad-host-range origin of replication (pBBR1) and mobilisation gene *mob*. This 4045 bp DNA fragment was amplified by PCR using primers P015_71101_f and P016_71101_r from plasmid pME6000 (2), followed by restriction digestion with PmeI and SbfI. Second,  the 2418 bp AscI-PmeI-flanked DNA fragment including the Gram positive replicon pCB102 and chloramphenicol resistance marker (*catP*) was replaced with a 805 bp DNA fragment containing solely *catP*, which was amplified by PCR using primers P017_CatP_f and P018_CatP_r from pMTL83141, followed by restriction digestion with AscI and PmeI. Third, the NotI restriction site in the *mob* gene locus was removed by conservative change using the QuikChange Site-Directed Mutagenesis Kit (Agilent Technologies) and primers P019_Qmob_f and P020_Qmob_r by following manufacturer's instructions. The resulting plasmid pMTL71101 contains the pBBR1 origin of replication and mobilisation gene *mob* (PmeI-SbfI module), the chloramphenicol resistance marker *catP* (FseI-PmeI module) and a multiple cloning site (SbfI-AscI module) (Supplementary Figure S1).

## Codon-optimised *P. alba ispS* gene encoding for isoprene synthase, 49 amino acids truncated at N-terminus

```
ATGGAAGCCCGCCGCTCGGCCAACTACGAGCCGAACTCGTGGGACTACGACTACCTGCTGTC
GTCGGACACCGACGAGTCGATCGAGGTGTACAAGGACAAGGCCAAGAAGCTGGAAGCCGAGG
TCCGCCGCGAGATCAACAACGAGAAGGCCGAGTTCCTGACGCTGCTGGAACTGATCGACAAC
GTGCAGCGCCTGGGCCTGGGCTACCGCTTCGAAAGCGACATCCGCGGCGCCCTGGACCGCTT
```

CGTGAGCAGCGGCGGCTTCGACGCCGTGACCAAGACCTCGCTGCATGGCACCGCGCTGTCGT
TCCGCCTGCTGCGCCAGCACGGCTTCGAGGTGTCGCAGGAAGCCTTCTCGGGCTTCAAGGAC
CAGAACGGCAACTTCCTGGAAAACCTGAAGGAAGATATCAAGGCCATCCTGTCGCTGTACGA
GGCCAGCTTCCTGGCGCTGGAAGGCGAGAACATCCTGGACGAGGCCAAGGTGTTCGCCATCT
CGCATCTGAAGGAACTGTCGGAGGAAAAGATCGGCAAGGAACTGGCCGAACAGGTGAACCAT
GCGCTGGAACTGCCGCTGCACCGCCGCACGCAGCGCCTGGAAGCCGTGTGGTCGATCGAAGC
CTACCGCAAGAAGGAAGACGCCAACCAGGTCCTGCTGGAACTGGCCATCCTGGACTACAACA
TGATCCAGTCGGTGTACCAGCGCGACCTGCGCGAAACCAGCCGCTGGTGGCGCCGCGTCGGC
CTGGCCACCAAGCTGCACTTCGCCCGCGACCGCCTGATCGAGTCGTTCTACTGGGCCGTGGG
CGTCGCCTTCGAGCCGCAGTATTCGGACTGCCGCAACTCGGTGGCCAAGATGTTCAGCTTCG
TGACCATCATCGACGACATCTACGACGTGTACGGCACCCTGGACGAACTGGAACTGTTCACC
GACGCGGTGGAACGCTGGGACGTGAACGCCATCAACGACCTGCCGGACTATATGAAGCTGTG
CTTCCTGGCGCTGTACAACACCATCAACGAGATCGCCTACGACAATCTGAAGGACAAGGGCG
AAAATATCCTGCCGTACCTGACCAAGGCCTGGGCCGACCTGTGCAACGCCTTCCTGCAGGAA
GCGAAGTGGCTGTATAACAAGTCGACCCCGACCTTCGACGACTACTTCGGCAACGCGTGGAA
GTCGTCGTCGGGCCCGCTGCAGCTGGTGTTCGCGTACTTCGCCGTGGTGCAGAACATCAAGA
AGGAAGAGATCGAGAACCTGCAGAAGTACCACGACACCATCTCGCGCCCGTCGCACATCTTC
CGCCTGTGCAATGACCTGGCCTCGGCCTCGGCGGAAATCGCCCGCGGCGAAACCGCCAACAG
CGTGTCGTGCTACATGCGCACCAAGGGCATCTCGGAAGAACTGGCGACCGAGTCGGTGATGA
ACCTGATCGACGAAACCTGGAAGAAGATGAACAAGGAAAAGCTGGGCGGCTCGCTGTTCGCC
AAGCCGTTCGTGGAAACCGCGATCAATCTGGCCCGCCAGTCGCACTGCACCTACCACAACGG
CGACGCGCACACCTCGCCGGATGAGCTGACCCGCAAGCGCGTGCTGAGCGTGATCACCGAGC
CGATCCTGCCGTTCGAGCGCTGA

## SUPPLEMENTARY TABLES

**Supplementary Table S1.** Oligonucleotide primers used in this study.

| Primer | Sequence 5' to 3' |
|---|---|
| P001 | atatggtaccaaattaagcagaaggccatcctgacggatggcctttttgcgtttcttaaggctgagggaaagtctagacgattcattaatgcagctggc |
| P002 | aattaccggtaaacaacagataaaacgaaaggcccgaaggcctttcgttttatttgatgctagctgaggagacatctagaaaggaagggaagaaagcg |
| P003 | gggaaaguacccaaaaattcatccttctcg |
| P004 | ggagacauggatccaagcttacttgtacagctcgtccatgc |
| P005_p0_1_r | attgtctcuctgccgtcactattcg |
| P005_p0_1-sd_r | attgagagugagccgtcactattcgaaccg |
| P005_p0_1+SD_r | agtgaattcugacatttgagtccattgttgcc |
| P005_p0_2_r | attgtctcuctgccgacatttgagtccattgttgcc |
| P005_p1_r | attataattgutatccgctcacaaagcaaataaatttttttatcgattaatccgcctcggcactgc |
| P005_p2_r | aaagtcagucctagggtagtcactatcatcgtgcaagttcgtttttaatccgcctcggcactgc |
| P005_p3_r | attcattauacgagccgatgattaattgtcaacagctcatcgattaatccgcctcggcactgc |
| P005_p4_r | agtatattcuaaatttccacctgtgtcaataacggttttatcgattaatccgcctcggcactgc |
| P005_p5_r | acgagaauttgaagcgtttagcaaatgaattttttaatcgattaatccgcctcggcactgc |
| P005_p6_r | attctattauacagaaaaattttcctgaaagcaaataaatttttttatcgattaatccgcctcggcactgc |
| P005_p7_r | aggtctataauaccacaatagtatcagtatgtaaacaactttctgaaatttttttatcgattaatccgcctcggcactgc |
| P005_p8_r | atgaatctautatatcgccgcaagggataaaaagcaaataaatttttttatcgattaatccgcctcggcactgc |
| P005_p9_r | atgaatctautataggtacaaaaagatgcgaagtcaatactctttttatcgattaatccgcctcggcactgc |
| P005_p10_r | atcttcaauattgagtggcacggttaagctccaaaatgacgatcgattaatccgcctcggcactgc |
| P005_p11_r | acgttaaauctaggtacaaaaagatgcgaaagcaaataaatttttttatcgattaatccgcctcggcactgc |
| P005_p12_r | agcacaauacctaggactgagctagccgtcaaatcgattaatccgcctcggcactgc |
| P005_p13_r | acacattauacgagccggatgattaaaagggaaatcgattaatccgcctcggcactgc |
| P005_p14_r | attcgtcccuccacacatcctaggagccggatgattaattgtcaaatcgattaatccgcctcggcactgc |
| P005_p15_r | attcaggtugctagcacagtacctaggactgagctagctgtcaaatcgattaatccgcctcggcactgc |
| P005_p16_r | attaagcuagcactgtacctaggactgagctagccgtcaaatcgattaatccgcctcggcactgc |

| | |
|---|---|
| P005_p17_r | atgaaataautatgggtacaaaaagatgcgatgtcaaatcgattaatccgcctcggcactgc |
| P005_p18_r | atgaaataautatgcctataggttagactttatgtcaaatcgattaatccgcctcggcactgc |
| P005_p19_r | acgttaaauctatcgccgcaagggataaatgtcaaatcgattaatccgcctcggcactgc |
| P005_p20_r | atgaaataautatgggtacaaaaagatgcgaaagcaaataaatttttatcgattaatccgcctcggcactgc |
| P005_p21_r | actccacauactctacgagccggatgattaattgtcaaatcgattaatccgcctcggcactgc |
| P005_p22_r | attcttcgugctagcattatacctaggactgagctagctgtcaaatcgattaatccgcctcggcactgc |
| P005_p23_r | actatgaauctattatacctataggttagactttatgtcaaatcgattaatccgcctcggcactgc |
| P005_p24_r | atccacacautatacagaaaaattttcctgaagtcaatactctttttatcgattaatccgcctcggcactgc |
| P005_p25_r | atctcacaautccacacattatacgagccggatgattaattgtcaaatcgattaatccgcctcggcactgc |
| P006_p0_f | gggaaagugacggcagagagacaatc |
| P006_p0_1_f | agagacaaucaacatatggtgagc |
| P006_p0_1-sd_f | actctcaaucaacatatggtgagcaagg |
| P006_p0_1+SD_f | agaattcacuagtttaactttaagaaggagatatacatatggtgagcaagggc |
| P006_p0_2_f | agagacaaucaacatatggtgagc |
| P006_p1_f | acaattataauagaattcactagtttaactttaagaaggagatatacatatggtgagcaagggc |
| P006_p2_f | actgacttucaaatcgatagcaagcaggtctctcgaggaagccaatcatggtgagcaagggcgag |
| P006_p3_f | ataatgaautcactagtttaactttaagaaggagatatacatatggtgagcaagggc |
| P006_p4_f | agaatatacutgaattcactagtttaactttaagaaggagatatacatatggtgagcaagggc |
| P006_p5_f | attctcguataatagaattcactagtttaactttaagaaggagatatacatatggtgagcaagggc |
| P006_p6_f | ataatagaautcactagtttaactttaagaaggagatatacatatggtgagcaagggc |
| P006_p7_f | attatagaccuatgaattcactagtttaactttaagaaggagatatacatatggtgagcaagggc |
| P006_p8_f | atagattcaucttagaattcactagtttaactttaagaaggagatatacatatggtgagcaagggc |
| P006_p9_f | atagattcautgctagaattcactagtttaactttaagaaggagatatacatatggtgagcaagggc |
| P006_p10_f | attgaagauagaattcactagtttaactttaagaaggagatatacatatggtgagcaagggc |
| P006_p11_f | atttaacguatcccgagaattcactagtttaactttaagaaggagatatacatatggtgagcaagggc |
| P006_p12_f | attgtgcuagccgtcggaattcactagtttaactttaagaaggagatatacatatggtgagcaagggc |
| P006_p13_f | ataatgtguggagacttgaattcactagtttaactttaagaaggagatatacatatggtgagcaagggc |
| P006_p14_f | agggacgaautcactagtttaactttaagaaggagatatacatatggtgagcaagggc |

| | |
|---|---|
| P006_p15_f | aacctgaautcactagtttaactttaagaaggagatatacatatggtgagcaagggc |
| P006_p16_f | agcttaaugaattcactagtttaactttaagaaggagatatacatatggtgagcaagggc |
| P006_p17_f | attatttcaugcgtcgaattcactagtttaactttaagaaggagatatacatatggtgagcaagggc |
| P006_p18_f | attatttcauccgacgaattcactagtttaactttaagaaggagatatacatatggtgagcaagggc |
| P006_p19_f | atttaacguatgacgggaattcactagtttaactttaagaaggagatatacatatggtgagcaagggc |
| P006_p20_f | attatttcaucttctgaattcactagtttaactttaagaaggagatatacatatggtgagcaagggc |
| P006_p21_f | atgtggaguatcgaattcactagtttaactttaagaaggagatatacatatggtgagcaagggc |
| P006_p22_f | acgaagaautcactagtttaactttaagaaggagatatacatatggtgagcaagggc |
| P006_p23_f | attcatagugagaattcactagtttaactttaagaaggagatatacatatggtgagcaagggc |
| P006_p24_f | atgtgtggaugttcagaattcactagtttaactttaagaaggagatatacatatggtgagcaagggc |
| P006_p25_f | attgtgagaugaggaattcactagtttaactttaagaaggagatatacatatggtgagcaagggc |
| P007_rbs0_r | accattacatauagaggaaggccgtcactattcgaaccg |
| P007_rbs1_r | atatctccutcgccgtcactattcgaaccg |
| P007_rbs2_r | aagtagtccuccgccgtcactattcgaaccg |
| P007_rbs3_r | agtctgtccuccgccgtcactattcgaaccg |
| P007_rbs4_r | acttactccuccgccgtcactattcgaaccg |
| P007_rbs5_r | atccttgttcucctttgccgtcactattcgaaccg |
| P007_rbs6_r | actatctccutcgccgtcactattcgaaccg |
| P007_rbs7_r | accttatccuccgccgtcactattcgaaccg |
| P007_rbs8_r | atccttgtccucctttgccgtcactattcgaaccg |
| P007_rbs9_r | acctcctucgccgtcactattcgaaccg |
| P007_rbs10_r | atttacctccutcggccgtcactattcgaaccg |
| P007_rbs11_r | aacctcctucgccgtcactattcgaaccg |
| P007_rbs12_r | attaacctccutcgccgtcactattcgaaccg |
| P007_rbs13_r | aacctcctucgccgtcactattcgaaccg |
| P007_rbs14_r | aacctcctucgccgtcactattcgaaccg |
| P007_rbs20_r | attgtctctcucgccgtcactattcgaaccg |
| P007_rbs21_r | attcctttucctccgccgtcactattcgaaccg |

| | |
|---|---|
| P007_rbs22_r | attctattcctccgccgtcactattcgaaccg |
| P007_rbs23_r | atggtctccuccgccgtcactattcgaaccg |
| P007_rbs24_r | acttcctccuttgccgtcactattcgaaccg |
| P007_rbs25_r | accacctccutcgccgtcactattcgaaccg |
| P007_rbs26_r | acgtcctccutagccgtcactattcgaaccg |
| P007_rbs27_r | atctcctutgccgtcactattcgaaccg |
| P007_rbs28_r | atatctccuttgccgtcactattcgaaccg |
| P007_rbs29_r | atatctcctucgccgtcactattcgaaccg |
| P007_rbs30_r | acactccutcgccgtcactattcgaaccg |
| P007_rbs31_r | acttgatccuccgccgtcactattcgaaccg |
| P007_rbs32_r | atatttccuccgccgtcactattcgaaccg |
| P007_SL/rbs_r | attatttcuagagggaaaccgttgtggtctcccgccgtcactattcgaaccg |
| P007_(A/U)$_{12}$/rbs1_r | atatctccutcttaaaagatcttttgccgtcactattcgaaccg |
| P007_(A/U)$_{12}$/rbs2_r | aagtagtccuccttaaaagatcttttgccgtcactattcgaaccg |
| P007_(A/U)$_{12}$/rbs8_r | atccttgtccucctttttaaaagatcttttgccgtcactattcgaaccg |
| P007_(A/U)$_{9}$/rbs1_r | atatctccutcaaagatcttttgccgtcactattcgaaccg |
| P007_(A/U)$_{6}$/rbs1_r | atatctccutcgatcttttgccgtcactattcgaaccg |
| P007_(A/U)$_{4}$/rbs1_r | atatctccutccttttgccgtcactattcgaaccg |
| P008_rbs0_f | atatgtaatggugagcaagggcgaggagct |
| P008_rbs1_f | aggagatauacatatggtgagcaagggcgag |
| P008_rbs2_f | aggactactugagatggtgagcaagggcgag |
| P008_rbs3_f | aggacagacuatggtgagcaagggcgag |
| P008_rbs4_f | aggagtaaguatggtgagcaagggcgag |
| P008_rbs5_f | agaacaaggauggtgagcaagggcgag |
| P008_rbs6_f | aggagataguaatggtgagcaagggcgag |
| P008_rbs7_f | aggataagguatggtgagcaagggcgag |
| P008_rbs8_f | aggacaaggauggtgagcaagggcgag |

| | |
|---|---|
| P008_rbs9_f | aaggagguaagatggtgagcaagggcgag |
| P008_rbs10_f | aggaggtaaauaatggtgagcaagggcgag |
| P008_rbs11_f | aaggaggtuaacatggtgagcaagggcgag |
| P008_rbs12_f | aggaggttaauacatggtgagcaagggcgag |
| P008_rbs13_f | aaggaggtucatatggtgagcaagggcgag |
| P008_rbs14_f | aaggaggtuaacatatggtgagcaagggcgag |
| P008_rbs20_f | agagagacaaucaacatatggtgagcaagggcgag |
| P008_rbs21_f | aaaaggaauggtgagcaagggcgag |
| P008_rbs22_f | aaatagaauggtgagcaagggcgag |
| P008_rbs23_f | aggagaccauatggtgagcaagggcgag |
| P008_rbs24_f | aggaggaaguatggtgagcaagggcgag |
| P008_rbs25_f | aggaggtgguatggtgagcaagggcgag |
| P008_rbs26_f | aggaggacguatggtgagcaagggcgag |
| P008_rbs27_f | aaggagauttagatggtgagcaagggcgag |
| P008_rbs28_f | aggagatauagatggtgagcaagggcgag |
| P008_rbs29_f | aaggagatauagatggtgagcaagggcgag |
| P008_rbs30_f | aggagtgugtaatggtgagcaagggcgag |
| P008_rbs31_f | aggatcaagutgatggtgagcaagggcgag |
| P008_rbs32_f | aggaaatauaagatggtgagcaagggcgag |
| P008_SL/rbs1_f | agaaataautttggaaggagatatacatatggtgagcaagggcgag |
| P008_SL/rbs2_f | agaaataautttgggaggactacttgagatggtgagcaagggcgag |
| P008_SL/rbs8_f | agaaataautttgaaaggaggacaaggatggtgagcaagggcgag |
| P009_ispS_f | gacggcgaaggagatatacatatgg |
| P010_ispS_r | tatacttaagtctagattcagcgctcgaacgg |
| P011_16S_f | agagtttgatcctggctcag |
| P012_16S_r | ttaccgcggctgctggcac |
| P013_yfp_f | agaagaacggcatcaaggtg |
| P014_yfp_r | gaactccagcaggaccatgt |

| | |
|---|---|
| P015_71101_f | tatagtttaaacgtgctacgcctgaataagtg |
| P016_71101_r | tatacctgcaggctgaggtctgcctcgtgaag |
| P017_CatP_f | ttatggcgcgccggccggccagtgggcaagttgaaaaattcac |
| P018_CatP_r | aggtttaaacttagggtaacaaaaaacaccgtatttctac |
| P019_Qmob_f | cccgcgaccagaccacgtttgccgccgctgtggccgatctagggctgc |
| P020_Qmob_r | gcagccctagatcggccacagcggcggcaaacgtggtctggtcgcggg |
| EH007_f | atttctagagggaaaccgttgtggtctccctacgaccagtctaaaaagcgcct |
| EH008_r | atcgacgtcttaatctttctgcgaattgagatgacgc |
| EH011_f | aatccaagcgtttaaacggaggcagacaaggtatagggc |
| EH012_r | tctgcctccgtttaaacgcttggattctcaccaataaaaaacgc |
| EH016_r | cgaaaggctcagtcgaaagactgggcctttcgttttatgacgtcttatgacaacttgacggctacatcattc |
| EH025_f | tcagaaggccatcctgacggatggccttttggcgcgccaggccggccgata |
| EH026_r | catacgagccggaagcataaagtgtaaagccgatcgttgttgacactctatcattgatagagttattttaccacgggagaccacaacggtttcc |
| EH027_f | tacactttatgcttccggctcgtatgttgtgtggaattgtgagcggataacaatttcacacaggaaacagctatgacaaagttgcagccgaat |
| EH028_r | ggccatccgtcaggatggccttctgacgtctcaatcgtcaccctttctcgg |
| EH048_f | gacgtcagaaggccatcct |
| EH055_r | atccgtcaggatggccttctgacgtcttattcctgtgtccgggtcacg |
| EH056_f | tacactttatgcttccggctcgtatgttgtgtggaattgtgagcggataacaatttcacacaggaaacagctatgcctctgacagacaccc |
| EH057_r | tacgagccggaagcataaagtgtaaagccgatcggcttcacaaccgcacttgatttaatagaccataccgtctattatttctgggggagaccacaacggtttcc |
| EH109_r | tttaagaaggagatatacatatggcgagtagc |
| EH111_r | atccgtcaggatggccttctgacgtcctagcgcttgaatttcgcgtac |
| EH112_f | ccggctcgtatgttgtgtggaattgtgagcggataacaatttcacacaggaaacagctatggtgatcatgagtccaaagagaa |
| EH113_r | acaattccacacaacatacgagccggaagcataaagtgtaaagccgatcgaaatcataaaaaatttatttgctttgtgagcg |
| EH114_f | atgtatatctccttcttaaagttaaacaaaattatttctagtaac |
| EH310_f | gctactcgccatatgtatatctccttcttaaaagatcttttgaattcccaaaaaaacgggtatggagaaac |

**Supplementary Table S2.** Plasmids used and generated in this study.

| Plasmid | Characteristic | Reference or source |
|---|---|---|
| pMTL83141 | $Cm^r$, ColE1 RNA II, CatP, *lacZ, pCB102* | (1) |
| pME6000 | $Tet^r$, pBBR1 ori, *lacZ, mob*$^+$ | (2) |
| pBBR1MCS-2-P*phaC-eyfp-c1* | $Kan^r$; broad host range vector used as a backbone and to amplify the eyfp gene | (3) |
| pGEM-T | $Amp^r$; *E. coli* vector used to amplify the *lacZ* gene for blue/white colony screening | Promega |
| pEH006 | $Cm^r$; $P_{araC}$-*araC*-$T_{rrnB1}$ and $P_{araBAD}$-T7sl-*Ec*RBS-*rfp*-$T_{dbl}$ | (4) |
| pJOE7784.1 | $Kan^r$; vector used to amplify $P_{rhaSR}$-*rhaSR* and $P_{rhaBAD}$ | (5) |
| pJOE7801.1 | $Kan^r$; vector used to amplify *tetR* | (5) |
| pMTL71101 | $Cm^r$; broad host range modular vector used as a backbone | This study |
| pBBR1MCS-2-USER | $Kan^r$; broad host range vector with USER cassette | This study |
| pMTL71107 | $Cm^r$; broad host range modular vector with USER cassette | This study |
| pBBR1MCS-2-$P_0$ | $Kan^r$; $P_0$-*eyfp*, assembled from pBBR1MCS-2-USER1 and PCR fragments generated by PCR using primer pair P006_P0_f-P004 from pBBR1MCS-2-PphaC-eyfp-c1 | This study |
| pBBR1MCS-2-$P_{0\_1}$ | $Kan^r$; $P_{0\_1}$-*eyfp*, assembled from pBBR1MCS-2-USER1 and PCR fragments generated by PCR using primer pairs P003-P005_P0_1_r and P006_P0_1_f-P004 from pBBR1MCS-2-PphaC-eyfp-c1 | This study |
| pBBR1MCS-2-$P_{0\_1\text{-}sd}$ | $Kan^r$; $P_{0\_1\text{-}sd}$-*eyfp*, assembled as above using corresponding primers as described in *Material and Methods* | This study |
| pBBR1MCS-2-$P_{0\_1+SD}$ | $Kan^r$; $P_{0\_1+SD}$-*eyfp*, assembled as above using corresponding primers as described in *Material and Methods* | This study |

| | | |
|---|---|---|
| pBBR1MCS-2-$P_{0\_2}$ | $Kan^r$; $P_{0\_2}$-*eyfp*, assembled as above using corresponding primers as described in *Material and Methods* | This study |
| pBBR1MCS-2-$P_{1}$ | $Kan^r$; $P_{1}$-*eyfp*, assembled as above using corresponding primers as described in *Material and Methods* | This study |
| pBBR1MCS-2-$P_{2}$ | $Kan^r$; $P_{2}$-*eyfp*, assembled as above using corresponding primers as described in *Material and Methods* | This study |
| pBBR1MCS-2-$P_{3}$ | $Kan^r$; $P_{3}$-*eyfp*, assembled as above using corresponding primers as described in *Material and Methods* | This study |
| pBBR1MCS-2-$P_{4}$ | $Kan^r$; $P_{4}$-*eyfp*, assembled as above using corresponding primers as described in *Material and Methods* | This study |
| pBBR1MCS-2-$P_{5}$ | $Kan^r$; $P_{5}$-*eyfp*, assembled as above using corresponding primers as described in *Material and Methods* | This study |
| pBBR1MCS-2-$P_{6}$ | $Kan^r$; $P_{6}$-*eyfp*, assembled as above using corresponding primers as described in *Material and Methods* | This study |
| pBBR1MCS-2-$P_{7}$ | $Kan^r$; $P_{7}$-*eyfp*, assembled as above using corresponding primers as described in *Material and Methods* | This study |
| pBBR1MCS-2-$P_{8}$ | $Kan^r$; $P_{8}$-*eyfp*, assembled as above using corresponding primers as described in *Material and Methods* | This study |
| pBBR1MCS-2-$P_{9}$ | $Kan^r$; $P_{9}$-*eyfp*, assembled as above using corresponding primers as described in *Material and Methods* | This study |
| pBBR1MCS-2-$P_{10}$ | $Kan^r$; $P_{10}$-*eyfp*, assembled as above using corresponding primers as described in *Material and Methods* | This study |
| pBBR1MCS-2-$P_{11}$ | $Kan^r$; $P_{11}$-*eyfp*, assembled as above using corresponding primers as described in *Material and Methods* | This study |

| | | |
|---|---|---|
| pBBR1MCS-2-P$_{12}$ | Kan$^r$; P$_{12}$-*eyfp*, assembled as above using corresponding primers as described in *Material and Methods* | This study |
| pBBR1MCS-2-P$_{13}$ | Kan$^r$; P$_{13}$-*eyfp*, assembled as above using corresponding primers as described in *Material and Methods* | This study |
| pBBR1MCS-2-P$_{14}$ | Kan$^r$; P$_{14}$-*eyfp*, assembled as above using corresponding primers as described in *Material and Methods* | This study |
| pBBR1MCS-2-P$_{15}$ | Kan$^r$; P$_{15}$-*eyfp*, assembled as above using corresponding primers as described in *Material and Methods* | This study |
| pBBR1MCS-2-P$_{16}$ | Kan$^r$; P$_{16}$-*eyfp*, assembled as above using corresponding primers as described in *Material and Methods* | This study |
| pBBR1MCS-2-P$_{17}$ | Kan$^r$; P$_{17}$-*eyfp*, assembled as above using corresponding primers as described in *Material and Methods* | This study |
| pBBR1MCS-2-P$_{18}$ | Kan$^r$; P$_{18}$-*eyfp*, assembled as above using corresponding primers as described in *Material and Methods* | This study |
| pBBR1MCS-2-P$_{19}$ | Kan$^r$; P$_{19}$-*eyfp*, assembled as above using corresponding primers as described in *Material and Methods* | This study |
| pBBR1MCS-2-P$_{20}$ | Kan$^r$; P$_{20}$-*eyfp*, assembled as above using corresponding primers as described in *Material and Methods* | This study |
| pBBR1MCS-2-P$_{21}$ | Kan$^r$; P$_{21}$-*eyfp*, assembled as above using corresponding primers as described in *Material and Methods* | This study |
| pBBR1MCS-2-P$_{22}$ | Kan$^r$; P$_{22}$-*eyfp*, assembled as above using corresponding primers as described in *Material and Methods* | This study |
| pBBR1MCS-2-P$_{23}$ | Kan$^r$; P$_{23}$-*eyfp*, assembled as above using corresponding primers as described in *Material and Methods* | This study |

| | | |
|---|---|---|
| pBBR1MCS-2-$P_{24}$ | Kan$^r$; $P_{24}$-*eyfp*, assembled as above using corresponding primers as described in *Material and Methods* | This study |
| pBBR1MCS-2-$P_{25}$ | Kan$^r$; $P_{25}$-*eyfp*, assembled as above using corresponding primers as described in *Material and Methods* | This study |
| pMTL71101-$P_{0}$ | Cm$^r$; $P_{0}$-*eyfp*, assembled from pMTL71107 and PCR fragments generated by PCR using primer pair P006_P0_f-P004 from pBBR1MCS-2-PphaC-eyfp-c1 | This study |
| pMTL71101-$P_{0\_1}$ | Cm$^r$; $P_{0\_1}$-*eyfp*, assembled from pMTL71107 and PCR fragments generated by PCR using primer pairs P003-P005_P0_1_r and P006_P0_1_f-P004 from pBBR1MCS-2-PphaC-eyfp-c1 | This study |
| pMTL71101-$P_{0\_1\text{-}sd}$ | Cm$^r$; $P_{0\_1\text{-}sd}$-*eyfp*, assembled as above using corresponding primers as described in *Material and Methods* | This study |
| pMTL71101-$P_{0\_1+SD}$ | Cm$^r$; $P_{0\_1+SD}$-*eyfp*, assembled as above using corresponding primers as described in *Material and Methods* | This study |
| pMTL71101-$P_{0\_2}$ | Cm$^r$; $P_{0\_2}$-*eyfp*, assembled as above using corresponding primers as described in *Material and Methods* | This study |
| pMTL71101-$P_{1}$ | Cm$^r$; $P_{1}$-*eyfp*, assembled as above using corresponding primers as described in *Material and Methods* | This study |
| pMTL71101-$P_{2}$ | Cm$^r$; $P_{2}$-*eyfp*, assembled as above using corresponding primers as described in *Material and Methods* | This study |
| pMTL71101-$P_{3}$ | Cm$^r$; $P_{3}$-*eyfp*, assembled as above using corresponding primers as described in *Material and Methods* | This study |
| pMTL71101-$P_{4}$ | Cm$^r$; $P_{4}$-*eyfp*, assembled as above using corresponding primers as described in *Material and Methods* | This study |
| pMTL71101-$P_{5}$ | Cm$^r$; $P_{5}$-*eyfp*, assembled as above using corresponding primers as described in *Material and Methods* | This study |

| | | |
|---|---|---|
| pMTL71101-$P_{6}$ | $Cm^r$; $P_{6}$-*eyfp*, assembled as above using corresponding primers as described in *Material and Methods* | This study |
| pMTL71101-$P_{7}$ | $Cm^r$; $P_{7}$-*eyfp*, assembled as above using corresponding primers as described in *Material and Methods* | This study |
| pMTL71101-$P_{8}$ | $Cm^r$; $P_{8}$-*eyfp*, assembled as above using corresponding primers as described in *Material and Methods* | This study |
| pMTL71101-$P_{9}$ | $Cm^r$; $P_{9}$-*eyfp*, assembled as above using corresponding primers as described in *Material and Methods* | This study |
| pMTL71101-$P_{10}$ | $Cm^r$; $P_{10}$-*eyfp*, assembled as above using corresponding primers as described in *Material and Methods* | This study |
| pMTL71101-$P_{11}$ | $Cm^r$; $P_{11}$-*eyfp*, assembled as above using corresponding primers as described in *Material and Methods* | This study |
| pMTL71101-$P_{12}$ | $Cm^r$; $P_{12}$-*eyfp*, assembled as above using corresponding primers as described in *Material and Methods* | This study |
| pMTL71101-$P_{13}$ | $Cm^r$; $P_{13}$-*eyfp*, assembled as above using corresponding primers as described in *Material and Methods* | This study |
| pMTL71101-$P_{14}$ | $Cm^r$; $P_{14}$-*eyfp*, assembled as above using corresponding primers as described in *Material and Methods* | This study |
| pMTL71101-$P_{15}$ | $Cm^r$; $P_{15}$-*eyfp*, assembled as above using corresponding primers as described in *Material and Methods* | This study |
| pMTL71101-$P_{16}$ | $Cm^r$; $P_{16}$-*eyfp*, assembled as above using corresponding primers as described in *Material and Methods* | This study |
| pMTL71101-$P_{17}$ | $Cm^r$; $P_{17}$-*eyfp*, assembled as above using corresponding primers as described in *Material and Methods* | This study |

| | | |
|---|---|---|
| pMTL71101-$P_{18}$ | $Cm^r$; $P_{18}$-*eyfp*, assembled as above using corresponding primers as described in *Material and Methods* | This study |
| pMTL71101-$P_{19}$ | $Cm^r$; $P_{19}$-*eyfp*, assembled as above using corresponding primers as described in *Material and Methods* | This study |
| pMTL71101-$P_{20}$ | $Cm^r$; $P_{20}$-*eyfp*, assembled as above using corresponding primers as described in *Material and Methods* | This study |
| pMTL71101-$P_{21}$ | $Cm^r$; $P_{21}$-*eyfp*, assembled as above using corresponding primers as described in *Material and Methods* | This study |
| pMTL71101-$P_{22}$ | $Cm^r$; $P_{22}$-*eyfp*, assembled as above using corresponding primers as described in *Material and Methods* | This study |
| pMTL71101-$P_{23}$ | $Cm^r$; $P_{23}$-*eyfp*, assembled as above using corresponding primers as described in *Material and Methods* | This study |
| pMTL71101-$P_{24}$ | $Cm^r$; $P_{24}$-*eyfp*, assembled as above using corresponding primers as described in *Material and Methods* | This study |
| pMTL71101-$P_{25}$ | $Cm^r$; $P_{25}$-*eyfp*, assembled as above using corresponding primers as described in *Material and Methods* | This study |
| pBBR1MCS-2-$RBS_{0}$ | $Kan^r$; $P_{phaC}$-$RBS_{0}$-*eyfp*, assembled from pBBR1MCS-2-USER1 and PCR fragments generated by PCR using primer pairs P003-P007_rbs0_r and P008_rbs0_f-P004 from pBBR1MCS-2-PphaC-eyfp-c1 | This study |
| pBBR1MCS-2-$RBS_{1}$ | $Kan^r$; $P_{phaC}$-$RBS_{1}$-*eyfp*, assembled as above using corresponding primers as described in *Material and Methods* | This study |
| pBBR1MCS-2-$RBS_{2}$ | $Kan^r$; $P_{phaC}$-$RBS_{2}$-*eyfp*, assembled as above using corresponding primers as described in *Material and Methods* | This study |

| | | |
|---|---|---|
| pBBR1MCS-2-RBS$_{3}$ | Kan$^{r}$; P$_{phaC}$-RBS$_{3}$-*eyfp*, assembled as above using corresponding primers as described in *Material and Methods* | This study |
| pBBR1MCS-2-RBS$_{4}$ | Kan$^{r}$; P$_{phaC}$-RBS$_{4}$-*eyfp*, assembled as above using corresponding primers as described in *Material and Methods* | This study |
| pBBR1MCS-2-RBS$_{5}$ | Kan$^{r}$; P$_{phaC}$-RBS$_{5}$-*eyfp*, assembled as above using corresponding primers as described in *Material and Methods* | This study |
| pBBR1MCS-2-RBS$_{6}$ | Kan$^{r}$; P$_{phaC}$-RBS$_{6}$-*eyfp*, assembled as above using corresponding primers as described in *Material and Methods* | This study |
| pBBR1MCS-2-RBS$_{7}$ | Kan$^{r}$; P$_{phaC}$-RBS$_{7}$-*eyfp*, assembled as above using corresponding primers as described in *Material and Methods* | This study |
| pBBR1MCS-2-RBS$_{8}$ | Kan$^{r}$; P$_{phaC}$-RBS$_{8}$-*eyfp*, assembled as above using corresponding primers as described in *Material and Methods* | This study |
| pBBR1MCS-2-RBS$_{9}$ | Kan$^{r}$; P$_{phaC}$-RBS$_{9}$-*eyfp*, assembled as above using corresponding primers as described in *Material and Methods* | This study |
| pBBR1MCS-2-RBS$_{10}$ | Kan$^{r}$; P$_{phaC}$-RBS$_{10}$-*eyfp*, assembled as above using corresponding primers as described in *Material and Methods* | This study |
| pBBR1MCS-2-RBS$_{11}$ | Kan$^{r}$; P$_{phaC}$-RBS$_{11}$-*eyfp*, assembled as above using corresponding primers as described in *Material and Methods* | This study |
| pBBR1MCS-2-RBS$_{12}$ | Kan$^{r}$; P$_{phaC}$-RBS1$_{2}$-*eyfp*, assembled as above using corresponding primers as described in *Material and Methods* | This study |
| pBBR1MCS-2-RBS$_{13}$ | Kan$^{r}$; P$_{phaC}$-RBS$_{13}$-*eyfp*, assembled as above using corresponding primers as described in *Material and Methods* | This study |
| pBBR1MCS-2-RBS$_{14}$ | Kan$^{r}$; P$_{phaC}$-RBS$_{14}$-*eyfp*, assembled as above using corresponding primers as described in *Material and Methods* | This study |

| | | |
|---|---|---|
| pBBR1MCS-2-RBS$_{20}$ | Kan$^r$; P$_{phaC}$-RBS$_{20}$-*eyfp*, assembled as above using corresponding primers as described in *Material and Methods* | This study |
| pBBR1MCS-2-RBS$_{21}$ | Kan$^r$; P$_{phaC}$-RBS$_{21}$-*eyfp*, assembled as above using corresponding primers as described in *Material and Methods* | This study |
| pBBR1MCS-2-RBS$_{22}$ | Kan$^r$; P$_{phaC}$-RBS$_{22}$-*eyfp*, assembled as above using corresponding primers as described in *Material and Methods* | This study |
| pBBR1MCS-2-RBS$_{23}$ | Kan$^r$; P$_{phaC}$-RBS$_{23}$-*eyfp*, assembled as above using corresponding primers as described in *Material and Methods* | This study |
| pBBR1MCS-2-RBS$_{24}$ | Kan$^r$; P$_{phaC}$-RBS$_{24}$-*eyfp*, assembled as above using corresponding primers as described in *Material and Methods* | This study |
| pBBR1MCS-2-RBS$_{25}$ | Kan$^r$; P$_{phaC}$-RBS$_{25}$-*eyfp*, assembled as above using corresponding primers as described in *Material and Methods* | This study |
| pBBR1MCS-2-RBS$_{26}$ | Kan$^r$; P$_{phaC}$-RBS$_{26}$-*eyfp*, assembled as above using corresponding primers as described in *Material and Methods* | This study |
| pBBR1MCS-2-RBS$_{27}$ | Kan$^r$; P$_{phaC}$-RBS$_{27}$-*eyfp*, assembled as above using corresponding primers as described in *Material and Methods* | This study |
| pBBR1MCS-2-RBS$_{28}$ | Kan$^r$; P$_{phaC}$-RBS$_{28}$-*eyfp*, assembled as above using corresponding primers as described in *Material and Methods* | This study |
| pBBR1MCS-2-RBS$_{29}$ | Kan$^r$; P$_{phaC}$-RBS$_{29}$-*eyfp*, assembled as above using corresponding primers as described in *Material and Methods* | This study |
| pBBR1MCS-2-RBS$_{30}$ | Kan$^r$; P$_{phaC}$-RBS$_{30}$-*eyfp*, assembled as above using corresponding primers as described in *Material and Methods* | This study |
| pBBR1MCS-2-RBS$_{31}$ | Kan$^r$; P$_{phaC}$-RBS$_{31}$-*eyfp*, assembled as above using corresponding primers as described in *Material and Methods* | This study |

| | | |
|---|---|---|
| pBBR1MCS-2-RBS$_{32}$ | Kan$^r$; P$_{phaC}$-RBS$_{32}$-*eyfp*, assembled as above using corresponding primers as described in *Material and Methods* | This study |
| pBBR1MCS-2-SL/RBS$_1$ | Kan$^r$; P$_{phaC}$-T7*g10* mRNA stemloop-RBS$_1$-*eyfp*, assembled as above using corresponding primers as described in *Material and Methods* | This study |
| pBBR1MCS-2-SL/RBS$_2$ | Kan$^r$; P$_{phaC}$-T7*g10* mRNA stemloop-RBS$_2$-*eyfp*, assembled as above using corresponding primers as described in *Material and Methods* | This study |
| pBBR1MCS-2-SL/RBS$_8$ | Kan$^r$; P$_{phaC}$-T7*g10* mRNA stemloop-RBS$_8$-*eyfp*, assembled as above using corresponding primers as described in *Material and Methods* | This study |
| pBBR1MCS-2-(A/U)$_4$/RBS$_1$ | Kan$^r$; P$_{phaC}$-(A/U)$_4$-RBS$_1$-*eyfp*, assembled as above using corresponding primers as described in *Material and Methods* | This study |
| pBBR1MCS-2-(A/U)$_6$/RBS$_1$ | Kan$^r$; P$_{phaC}$-(A/U)$_6$-RBS$_1$-*eyfp*, assembled as above using corresponding primers as described in *Material and Methods* | This study |
| pBBR1MCS-2-(A/U)$_9$/RBS$_1$ | Kan$^r$; P$_{phaC}$-(A/U)$_9$-RBS$_1$-*eyfp*, assembled as above using corresponding primers as described in *Material and Methods* | This study |
| pBBR1MCS-2-(A/U)$_{12}$/RBS$_1$ | Kan$^r$; P$_{phaC}$-(A/U)$_{12}$-RBS$_1$-*eyfp*, assembled as above using corresponding primers as described in *Material and Methods* | This study |
| pBBR1MCS-2-(A/U)$_{12}$/RBS$_2$ | Kan$^r$; P$_{phaC}$-(A/U)$_{12}$-RBS$_2$-*eyfp*, assembled as above using corresponding primers as described in *Material and Methods* | This study |
| pBBR1MCS-2-(A/U)$_{12}$/RBS$_8$ | Kan$^r$; P$_{phaC}$-(A/U)$_{12}$-RBS$_8$-*eyfp*, assembled as above using corresponding primers as described in *Material and Methods* | This study |
| pBBR1MCS-2-RBS$_1$-ispS | Kan$^r$; P$_{phaC}$-RBS$_1$-*ispS*, constructed by replacing *eyfp* with *ispS* in pBBR1MCS-2-RBS$_1$ as described in *Material and Methods* | This study |
| pBBR1MCS-2-RBS$_2$-ispS | Kan$^r$; P$_{phaC}$-RBS$_2$-*ispS*, constructed by replacing *eyfp* with *ispS* in pBBR1MCS-2-RBS$_2$ as described in *Material and Methods* | This study |

| | | |
|---|---|---|
| pBBR1MCS-2-RBS$_{5}$-ispS | Kan$^{r}$; $P_{phaC}$-RBS$_{5}$-*ispS*, constructed by replacing *eyfp* with *ispS* in pBBR1MCS-2-RBS$_{5}$ as described in *Material and Methods* | This study |
| pBBR1MCS-2-RBS$_{6}$-ispS | Kan$^{r}$; $P_{phaC}$-RBS$_{6}$-*ispS*, constructed by replacing *eyfp* with *ispS* in pBBR1MCS-2-RBS$_{6}$ as described in *Material and Methods* | This study |
| pBBR1MCS-2-RBS$_{8}$-ispS | Kan$^{r}$; $P_{phaC}$-RBS$_{8}$-*ispS*, constructed by replacing *eyfp* with *ispS* in pBBR1MCS-2-RBS$_{8}$ as described in *Material and Methods* | This study |
| pBBR1MCS-2-RBS$_{9}$-ispS | Kan$^{r}$; $P_{phaC}$-RBS$_{9}$-*ispS*, constructed by replacing *eyfp* with *ispS* in pBBR1MCS-2-RBS$_{9}$ as described in *Material and Methods* | This study |
| pBBR1MCS-2-RBS$_{13}$-ispS | Kan$^{r}$; $P_{phaC}$-RBS$_{13}$-*ispS*, constructed by replacing *eyfp* with *ispS* in pBBR1MCS-2-RBS$_{13}$ as described in *Material and Methods* | This study |
| pBBR1MCS-2-RBS$_{14}$-ispS | Kan$^{r}$; $P_{phaC}$-RBS$_{14}$-*ispS*, constructed by replacing *eyfp* with *ispS* in pBBR1MCS-2-RBS$_{14}$ as described in *Material and Methods* | This study |
| pBBR1MCS-2-RBS$_{20}$-ispS | Kan$^{r}$; $P_{phaC}$-RBS$_{20}$-*ispS*, constructed by replacing *eyfp* with *ispS* in pBBR1MCS-2-RBS$_{20}$ as described in *Material and Methods* | This study |
| pBBR1MCS-2-RBS$_{24}$-ispS | Kan$^{r}$; $P_{phaC}$-RBS$_{24}$-*ispS*, constructed by replacing *eyfp* with *ispS* in pBBR1MCS-2-RBS$_{24}$ as described in *Material and Methods* | This study |
| pBBR1MCS-2-RBS$_{25}$-ispS | Kan$^{r}$; $P_{phaC}$-RBS$_{25}$-*ispS*, constructed by replacing *eyfp* with *ispS* in pBBR1MCS-2-RBS$_{25}$ as described in *Material and Methods* | This study |
| pBBR1MCS-2-RBS$_{28}$-ispS | Kan$^{r}$; $P_{phaC}$-RBS$_{28}$-*ispS*, constructed by replacing *eyfp* with *ispS* in pBBR1MCS-2-RBS$_{28}$ as described in *Material and Methods* | This study |
| pBBR1MCS-2-RBS$_{31}$-ispS | Kan$^{r}$; $P_{phaC}$-RBS$_{31}$-*ispS*, constructed by replacing *eyfp* with *ispS* in pBBR1MCS-2-RBS$_{31}$ as described in *Material and Methods* | This study |
| pBBR1MCS-2-RBS$_{32}$-ispS | Kan$^{r}$; $P_{phaC}$-RBS$_{32}$-*ispS*, constructed by replacing *eyfp* with *ispS* in pBBR1MCS-2-RBS$_{32}$ as described in *Material and Methods* | This study |

| | | |
|---|---|---|
| pBBR1MCS-2-$P_{1}$-$RBS_{1}$ | $Kan^r$; $P_{1}$-$RBS_{1}$-*eyfp,* constructed by replacing $P_{phaC}$ with $P_{1}$ in pBBR1MCS-2-$RBS_{1}$ | This study |
| pBBR1MCS-2-$P_{1}$-$RBS_{2}$ | $Kan^r$; $P_{1}$-$RBS_{2}$-*eyfp,* constructed by replacing $P_{phaC}$ with $P_{1}$ in pBBR1MCS-2-$RBS_{2}$ | This study |
| pBBR1MCS-2-$P_{1}$-$RBS_{8}$ | $Kan^r$; $P_{1}$-$RBS_{8}$-*eyfp,* constructed by replacing $P_{phaC}$ with $P_{1}$ in pBBR1MCS-2-$RBS_{8}$ | This study |
| pBBR1MCS-2-$P_{rhaBAD}$-$RBS_{1}$ | $Kan^r$; $P_{rhaBAD}$-$RBS_{1}$-*eyfp,* constructed by replacing $P_{phaC}$ with $P_{rhaBAD}$ in pBBR1MCS-2-$RBS_{1}$ | This study |
| pBBR1MCS-2-$P_{rhaBAD}$-$RBS_{2}$ | $Kan^r$; $P_{rhaBAD}$-$RBS_{2}$-*eyfp,* constructed by replacing $P_{phaC}$ with $P_{rhaBAD}$ in pBBR1MCS-2-$RBS_{2}$ | This study |
| pBBR1MCS-2-$P_{rhaBAD}$-$RBS_{8}$ | $Kan^r$; $P_{rhaBAD}$-$RBS_{8}$-*eyfp,* constructed by replacing $P_{phaC}$ with $P_{rhaBAD}$ in pBBR1MCS-2-$RBS_{8}$ | This study |
| pBBR1MCS-2-$RBS_{1}$-rfp | $Kan^r$; $P_{phaC}$-$RBS_{1}$-*rfp,* constructed by replacing *eyfp* with *rfp* in pBBR1MCS-2-$RBS_{1}$ | This study |
| pBBR1MCS-2-$RBS_{2}$-rfp | $Kan^r$; $P_{phaC}$-$RBS_{2}$-*rfp*, constructed by replacing *eyfp* with *rfp* in pBBR1MCS-2-$RBS_{2}$ | This study |
| pBBR1MCS-2-$RBS_{8}$-rfp | $Kan^r$; $P_{phaC}$-$RBS_{8}$-*rfp*, *constructed by replacing eyfp with rfp* in pBBR1MCS-2-$RBS_{8}$ | This study |
| | | |
| pMTL71101-$RBS_{0}$ | $Cm^r$; $P_{phaC}$-$RBS_{0}$-*eyfp*, assembled from pMTL71107 and PCR fragments generated by PCR using primer pairs P003-P007_rbs0_r and P008_rbs0_f-P004 from pBBR1MCS-2-PphaC-eyfp-c1 | This study |
| pMTL71101-$RBS_{1}$ | $Cm^r$; $P_{phaC}$-$RBS_{1}$-*eyfp*, assembled as above using corresponding primers as described in *Material and Methods* | This study |
| pMTL71101-$RBS_{2}$ | $Cm^r$; $P_{phaC}$-$RBS_{2}$-*eyfp*, assembled as above using corresponding primers as described in *Material and Methods* | This study |
| pMTL71101-$RBS_{3}$ | $Cm^r$; $P_{phaC}$-$RBS_{3}$-*eyfp*, assembled as above using corresponding primers as described in *Material and Methods* | This study |
| pMTL71101-$RBS_{4}$ | $Cm^r$; $P_{phaC}$-$RBS_{4}$-*eyfp*, assembled as above using corresponding primers as described in *Material and Methods* | This study |
| pMTL71101-$RBS_{5}$ | $Cm^r$; $P_{phaC}$-$RBS_{5}$-*eyfp*, assembled as above using corresponding primers as described in *Material and Methods* | This study |
| pMTL71101-$RBS_{6}$ | $Cm^r$; $P_{phaC}$-$RBS_{6}$-*eyfp*, assembled as above using corresponding primers as described in *Material and Methods* | This study |

| | | |
|---|---|---|
| pMTL71101-$RBS_{7}$ | $Cm^r$; $P_{phaC}$-$RBS_{7}$-*eyfp*, assembled as above using corresponding primers as described in *Material and Methods* | This study |
| pMTL71101-$RBS_{8}$ | $Cm^r$; $P_{phaC}$-$RBS_{8}$-*eyfp*, assembled as above using corresponding primers as described in *Material and Methods* | This study |
| pMTL71101-$RBS_{9}$ | $Cm^r$; $P_{phaC}$-$RBS_{9}$-*eyfp*, assembled as above using corresponding primers as described in *Material and Methods* | This study |
| pMTL71101-$RBS_{10}$ | $Cm^r$; $P_{phaC}$-$RBS_{10}$-*eyfp*, assembled as above using corresponding primers as described in *Material and Methods* | This study |
| pMTL71101-$RBS_{11}$ | $Cm^r$; $P_{phaC}$-$RBS_{11}$-*eyfp*, assembled as above using corresponding primers as described in *Material and Methods* | This study |
| pMTL71101-$RBS_{12}$ | $Cm^r$; $P_{phaC}$-$RBS_{12}$-*eyfp*, assembled as above using corresponding primers as described in *Material and Methods* | This study |
| pMTL71101-$RBS_{13}$ | Cm; $P_{phaC}$-$RBS_{13}$-*eyfp*, assembled as above using corresponding primers as described in *Material and Methods* | This study |
| pMTL71101-$RBS_{14}$ | $Cm^r$; $P_{phaC}$-$RBS_{14}$-*eyfp*, assembled as above using corresponding primers as described in *Material and Methods* | This study |
| pMTL71101-$RBS_{20}$ | $Cm^r$; $P_{phaC}$-$RBS_{20}$-*eyfp*, assembled as above using corresponding primers as described in *Material and Methods* | This study |
| pMTL71101-$RBS_{21}$ | $Cm^r$; $P_{phaC}$-$RBS_{21}$-*eyfp*, assembled as above using corresponding primers as described in *Material and Methods* | This study |
| pMTL71101-$RBS_{22}$ | $Cm^r$; $P_{phaC}$-$RBS_{22}$-*eyfp*, assembled as above using corresponding primers as described in *Material and Methods* | This study |
| pMTL71101-$RBS_{23}$ | $Cm^r$; $P_{phaC}$-$RBS_{23}$-*eyfp*, assembled as above using corresponding primers as described in *Material and Methods* | This study |

| | | |
|---|---|---|
| pMTL71101-RBS$_{24}$ | Cm$^{r}$; P$_{phaC}$-RBS$_{24}$-*eyfp*, assembled as above using corresponding primers as described in *Material and Methods* | This study |
| pMTL71101-RBS$_{25}$ | Cm$^{r}$; P$_{phaC}$-RBS$_{25}$-*eyfp*, assembled as above using corresponding primers as described in *Material and Methods* | This study |
| pMTL71101-RBS$_{26}$ | Cm$^{r}$; P$_{phaC}$-RBS$_{26}$-*eyfp*, assembled as above using corresponding primers as described in *Material and Methods* | This study |
| pMTL71101-RBS$_{27}$ | Cm$^{r}$; P$_{phaC}$-RBS$_{27}$-*eyfp*, assembled as above using corresponding primers as described in *Material and Methods* | This study |
| pMTL71101-RBS$_{28}$ | Cm$^{r}$; P$_{phaC}$-RBS$_{28}$-*eyfp*, assembled as above using corresponding primers as described in *Material and Methods* | This study |
| pMTL71101-RBS$_{29}$ | Cm; P$_{phaC}$-RBS$_{29}$-*eyfp*, assembled as above using corresponding primers as described in *Material and Methods* | This study |
| pMTL71101-RBS$_{30}$ | Cm$^{r}$; P$_{phaC}$-RBS$_{30}$-*eyfp*, assembled as above using corresponding primers as described in *Material and Methods* | This study |
| pMTL71101-RBS$_{31}$ | Cm$^{r}$; P$_{phaC}$-RBS$_{31}$-*eyfp*, assembled as above using corresponding primers as described in *Material and Methods* | This study |
| pMTL71101-RBS$_{32}$ | Cm$^{r}$; P$_{phaC}$-RBS$_{32}$-*eyfp*, assembled as above using corresponding primers as described in *Material and Methods* | This study |
| pEH002 | Cm$^{r}$; P$_{rhaSR}$-*rhaSR*-T$_{rrnB1}$ and P$_{rhaBAD}$-T7sl-*Ec*RBS-*rfp*-T$_{dbl}$ from pJOE7784.1 cloned into pEH006 by AatII and XbaI sites | This study |
| pEH005 | Cm$^{r}$; P$_{lac}$-*tetR*-T$_{rrnB2}$ and P$_{tetA}$-T7sl-*Ec*RBS-*rfp*-T$_{dbl}$ from pJOE7801.1 assembled by NEBuilder | This study |
| pEH020 | Cm$^{r}$; P$_{lac}$-*acuR*-T$_{rrnB2}$ and P$_{acuR}$-T7sl-*Ec*RBS-*rfp*-T$_{dbl}$ from *R. sphaeroides* 2.4.1 genomic DNA assembled by NEBuilder | This study |

| | | |
|---|---|---|
| pEH040 | $Cm^r$; $P_{lac}$-*cymR*-$T_{rrnB2}$ and $P_{cmt}$-*Ec*RBS-*rfp*-$T_{dbl}$ from pNEW assembled by NEBuilder | This study |
| pEH002-ispS | $Cm^r$; $P_{rhaSR}$-*rhaSR*-$T_{rrnB1}$ and $P_{rhaBAD}$-T7sl-*Ec*RBS-*ispS*-$T_{dbl}$, *rfp* replaced with *ispS* in pEH002 through NdeI and BamHI restriction sites | This study |
| pEH005-ispS | $Cm^r$; $P_{lac}$-*tetR*-$T_{rrnB2}$ and $P_{tetA}$-T7sl-*Ec*RBS-*rfp*-$T_{dbl}$, *rfp* replaced with *ispS* in pEH002 through NdeI and BamHI restriction sites | This study |
| pEH020-ispS | $Cm^r$; $P_{lac}$-*acuR*-$T_{rrnB2}$ and $P_{acuR}$-T7sl-*Ec*RBS-*rfp*-$T_{dbl}$, *rfp* replaced with *ispS* in pEH002 through NdeI and BamHI restriction sites | This study |
| pEH040-ispS | $Cm^r$; $P_{lac}$-*cymR*-$T_{rrnB2}$ and $P_{cmt}$-*Ec*RBS-*rfp*-$T_{dbl}$, *rfp* replaced with *ispS* in pEH002 through NdeI and AflII restriction sites | This study |
| pEH176 | $Cm^r$; $P_{araC}$-*araC*-$T_{rrnB1}$ and $P_{araBAD}$-*Ec*RBS-*rfp*-$T_{dbl}$ from *E. coli* MG1655 genomic DNA cloned into pEH006 by AatII and NdeI sites | This study |

**Supplementary Table S3.** Hill function fitting parameters.

| Plasmid identifier | Inducer | Minimum RFP synthesis rate, $v_{min}$ ($s^{-1}$) | Maximum RFP synthesis rate, $v_{max}$ ($s^{-1}$) | Hill coefficient, *h* | Half-maximal RFP synthesis, $K_m$ (μM) |
|---|---|---|---|---|---|
| pEH002 | L-rhamnose | 0.0058 ± 0.0022 | 3.292 ± 0.032 | 3.88 ± 0.23 | 1057 ± 19 |
| pEH006 | L-arabinose | 0.0122 ± 0.0023 | 3.836 ± 0.085 | 1.70 ± 0.11 | 1116 ± 56 |
| pEH020 | acrylate | 0.0474 ± 0.0056 | 1.471 ± 0.035 | 1.03 ± 0.09 | 84.32 ± 8.08 |
| pEH040 | cumate | 0.0472 ± 0.0076 | 0.828 ± 0.011 | 1.56 ± 0.08 | 0.877 ± 0.029 |

**SUPPLEMENTARY FIGURES**

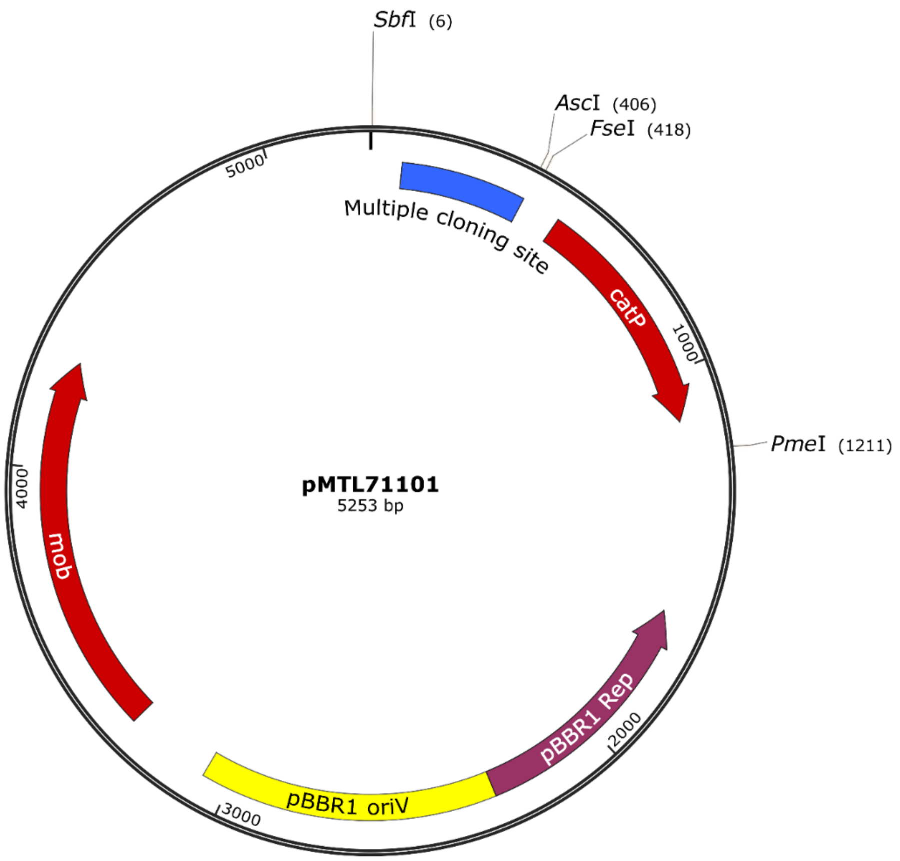


**Supplementary Figure S1.** Plasmid map of modular vector pMTL71101. Restriction endonucleases SbfI, AscI, FseI, and PmeI, flanking modules of the vector, are shown with their restriction site positions in the brackets. The pBBR1 origin of replication and mobilisation gene *mob* (PmeI-SbfI module), the chloramphenicol resistance marker *catP* (FseI-PmeI module) and a multiple cloning site (SbfI-AscI module) are highlighted.

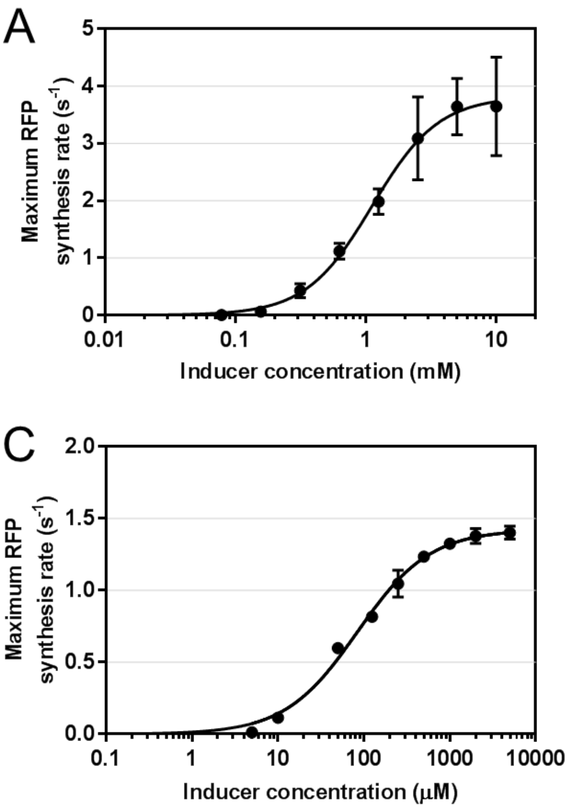


**Supplementary Figure S2.** Promotor activity of inducible systems. The normalised maximum rate of RFP synthesis was fit to the corresponding inducer concentration using a Hill function for the (A) L-arabinose-, (B) L-rhamnose-, (C) acrylate-, and (D) cumate-inducible systems. Error bars represent standard deviations of three biological replicates.

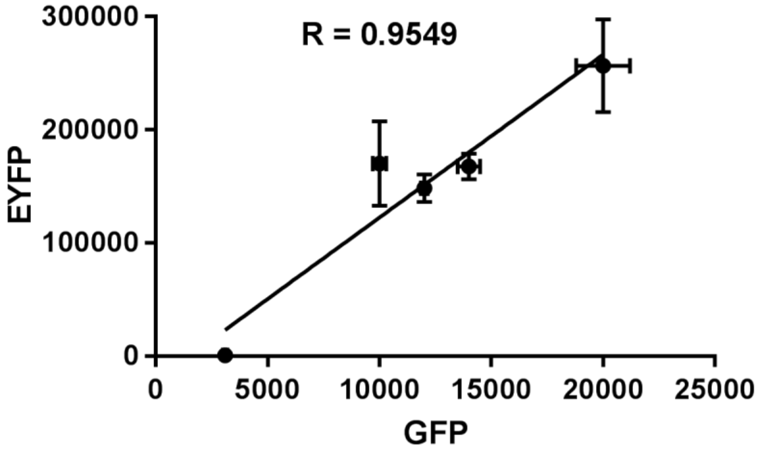


**Supplementary Figure S3.** Correlation between promoter activities, measured as normalised fluorescence arbitrary units (A.U.) using either GFP as reporter in previous study (6) and EYFP in this study. Core promoters derived from bacteriophage T5 and *C. necator* H16 (promoters $P_{T5}$, $P_{j5}$, $P_{h207}$, $P_{h25}$ and $P_{H16\_B1772}$ (6)) were used in analysis. In this study they comprise core part of promoters $P_1$, $P_4$, $P_5$, $P_6$, and $P_2$, respectively.

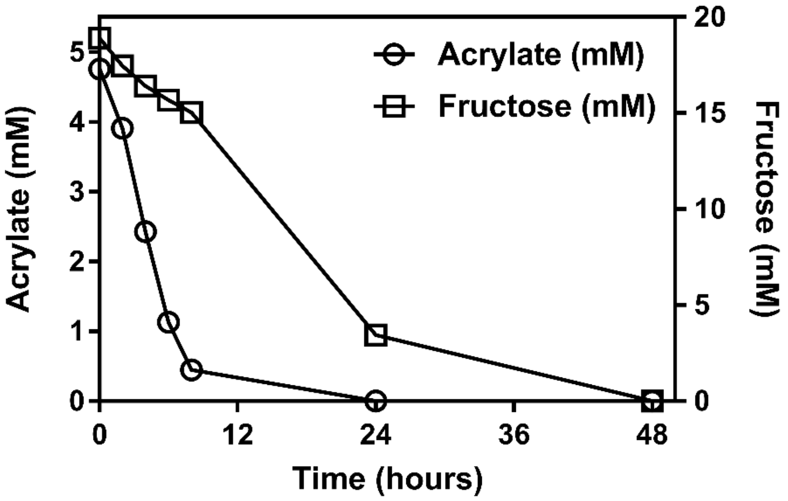


**Supplementary Figure S4.** Consumption of acrylate in *C. necator*. Acrylate was added at time zero. Consumption of acrylate and fructose was monitored using HPLC-UV in supernatant over the time course of 48 hours. The means of three biological replicates are presented. Error bars are too small to be visible.

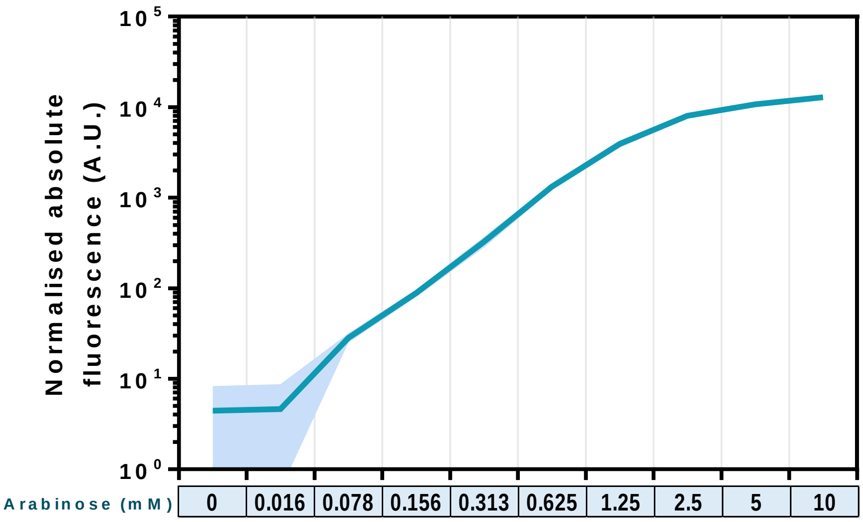


**Supplementary Figure S5.** Induction dynamics for the L-arabinose-inducible system lacking the stem-loop structure. Normalised absolute fluorescence of *C. necator* cells in exponential growth phase six hours after L-arabinose addition. The standard deviation of three biological replicates is illustrated as band.

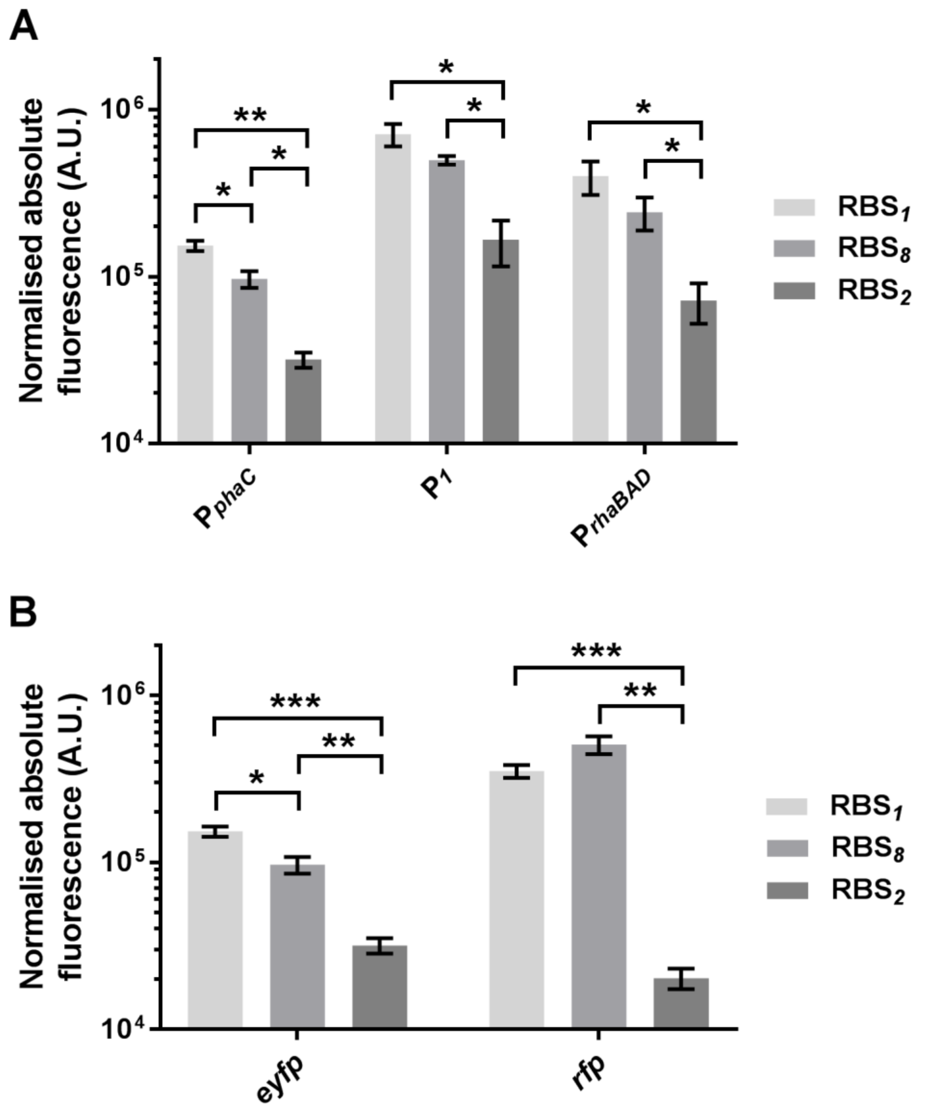


**Supplementary Figure S6.** Effect of sequence content on the strength of RBS. Normalised absolute fluorescence determined using plasmid constructs with promoters $P_{phaC}$, ($P_{0\_1}$), $P_{1}$, and $P_{rhaBAD}$ (A), and reporter genes *eyfp* and *rfp* (B) located upstream and downstream, respectively, of $RBS_{1}$, $RBS_{8}$ and $RBS_{2}$. Error bars represent standard deviation of three biological replicas. Asterisks indicate statistically significant difference between RBS strengths: *$p < 0.01$; **$p < 0.001$; and ***$p < 0.0001$ (unpaired t-test).

## REFERENCES


1. **Heap JT, Pennington OJ, Cartman ST, Minton NP.** 2009. A modular system for *Clostridium* shuttle plasmids. Journal of Microbiological Methods **78:**79-85.
2. **Maurhofer M, Reimmann C, Schmidli-Sacherer P, Heeb S, Haas D, Defago G.** 1998. Salicylic acid biosynthetic genes expressed in *Pseudomonas fluorescens* strain P3 improve the induction of systemic resistance in tobacco against tobacco necrosis virus. Phytopathology **88:**678-684.
3. **Pfeiffer D, Jendrossek D.** 2011. Interaction between poly(3-hydroxybutyrate) granule-associated proteins as revealed by two-hybrid analysis and identification of a new phasin in *Ralstonia eutropha* H16. Microbiology **157:**2795-2807.
4. **Hanko E, Minton N, Malys N.** 2017. Characterisation of a 3-hydroxypropionic acid-inducible system from *Pseudomonas putida* for orthogonal gene expression control in *Escherichia coli* and *Cupriavidus necator*. Scientific Reports **7:**1724.
5. **Hoffmann J, Altenbuchner J.** 2015. Functional characterization of the mannitol promoter of *Pseudomonas fluorescens* DSM 50106 and its application for a mannitol-inducible expression system for *Pseudomonas putida* KT2440. PLoS ONE **10**.
6. **Gruber S, Hagen J, Schwab H, Koefinger P.** 2014. Versatile and stable vectors for efficient gene expression in *Ralstonia eutropha* H16. Journal of Biotechnology **186:**74-82.
